# Translational imaging of TSPO reveals pronounced innate inflammation in human and murine CD8 T cell–mediated limbic encephalitis

**DOI:** 10.1126/sciadv.abq7595

**Published:** 2023-06-09

**Authors:** Marco Gallus, Wolfgang Roll, Andre Dik, Cristina Barca, Bastian Zinnhardt, Gordon Hicking, Christoph Mueller, Venu Narayanan Naik, Max Anstötz, Julia Krämer, Leoni Rolfes, Lydia Wachsmuth, Julika Pitsch, Karen M. J. van Loo, Saskia Räuber, Hideho Okada, Catriona Wimberley, Christine Strippel, Kristin S. Golombeck, Andreas Johnen, Stjepana Kovac, Catharina C. Groß, Philipp Backhaus, Robert Seifert, Jan Lewerenz, Rainer Surges, Christian E. Elger, Heinz Wiendl, Tobias Ruck, Albert J. Becker, Cornelius Faber, Andreas H. Jacobs, Jan Bauer, Sven G. Meuth, Michael Schäfers, Nico Melzer

**Affiliations:** ^1^ Department of Neurology Institute of Translational Neurology, University of Münster, Münster, Germany.; ^2^ Department of Neurosurgery, University of Münster, Münster, Germany.; ^3^ Department of Neurosurgery, University of California, San Francisco, San Francisco, CA, USA.; ^4^ Department of Nuclear Medicine, University of Münster, Münster, Germany.; ^5^ Department of Neurology, Medical Faculty, Heinrich Heine University of Düsseldorf, Düsseldorf, Germany.; ^6^ European Institute for Molecular Imaging, University of Münster, Münster, Germany.; ^7^ Biomarkers and Translational Technologies (BTT), Pharma Research and Early Development (pRED), F. Hoffmann-La Roche Ltd., Basel, Switzerland.; ^8^ Institute of Anatomy II, Medical Faculty, University Hospital Düsseldorf, Heinrich-Heine-University, Düsseldorf, Germany.; ^9^ Department of Clinical Radiology, University of Münster, Münster, Germany.; ^10^ Department of Epileptology, University of Bonn, Bonn, Germany.; ^11^ Section for Translational Epilepsy Research, Department of Neuropathology, University of Bonn, Bonn, Germany.; ^12^ Department of Epileptology and Neurology, RWTH Aachen University, Aachen, Germany.; ^13^ Edinburgh Imaging, University of Edinburgh, Edinburgh, UK.; ^14^ Department of Neurology, University of Ulm, Ulm, Germany.; ^15^ Department of Neuroimmunology, Centre for Brain Research, Medical University of Vienna, Vienna, Austria.

## Abstract

Autoimmune limbic encephalitis (ALE) presents with new-onset mesial temporal lobe seizures, progressive memory disturbance, and other behavioral and cognitive changes. CD8 T cells are considered to play a key role in those cases where autoantibodies (ABs) target intracellular antigens or no ABs were found. Assessment of such patients presents a clinical challenge, and novel noninvasive imaging biomarkers are urgently needed. Here, we demonstrate that visualization of the translocator protein (TSPO) with [^18^F]DPA-714-PET-MRI reveals pronounced microglia activation and reactive gliosis in the hippocampus and amygdala of patients suspected with CD8 T cell ALE, which correlates with FLAIR-MRI and EEG alterations. Back-translation into a preclinical mouse model of neuronal antigen-specific CD8 T cell–mediated ALE allowed us to corroborate our preliminary clinical findings. These translational data underline the potential of [^18^F]DPA-714-PET-MRI as a clinical molecular imaging method for the direct assessment of innate immunity in CD8 T cell–mediated ALE.

## Teaser

Imaging of the translocator protein detects innate inflammation in human and murine CD8 T cell-mediated limbic encephalitis.

## INTRODUCTION

Mesial temporal lobe epilepsy (mTLE) is a common adult focal epilepsy syndrome. Recent data have demonstrated limbic encephalitis with adaptive and concomitant innate autoimmune inflammation predominantly affecting the allocortex of the amygdala and hippocampus as a major cause of adult mTLE development (*1*–*3*). Autoimmune limbic encephalitis (ALE) usually presents with new onset of acute mesial temporal lobe seizures, progressive memory disturbance, and a variety of other behavioral and cognitive changes. Occasionally, development of hippocampal sclerosis (HS) might result in chronic pharmacoresistant mTLE (*4*, *5*).

CD8 T cells are deemed to be pathogenic in human ALE due to their strong clonal expansion, parenchymal localization in close spatial proximity to neurons, and granular expression and release of CD8 effector molecules towards targeted neurons (*6*–*11*). In addition to CD8 T cells, activated microglia and macrophages, partially forming nodules, can be detected in the brain parenchyma of patients with ALE (*10*). In some patients suffering from ALE, specific autoantibodies (AABs) are found in the serum and cerebrospinal fluid (CSF) (*12*, *13*). These AABs bind to either intracellular or plasma membrane neural antigens and illustrate the presence of an adaptive neural autoimmune reaction (*14*, *15*). Where AABs target intracellular neuronal antigens, CD8 T cells are considered to play a major pathogenic role in the development of neuronal dysfunction and cell death, as these antigens are initially not accessible to antibodies. Subsequent release of intracellular antigens may then initiate B cell responses and AAB production (*8*, *9*, *16*–*19*). Nevertheless, a pathogenic role of CD8 T cells has also been suggested when the AAB target antigen is (transiently) localized in the plasma membrane or remains elusive (*6*, *7*, *20*, *21*). Consistently, in cases of long-standing pharmacoresistant mTLE with HS of unknown origin, increased infiltrates with predominance of CD8 T cells accompanied by activated microglia and macrophages have been described mainly in the hippocampal cornu ammonis (CA) 1 region (*22*–*27*). These infiltrates correlate with the extent of hippocampal neuronal loss suggesting a pathogenic role of CD8 T cells and microglia/macrophages in driving long-standing epileptogenic neurodegeneration in these cases (*25*, *26*, *28*). Consistent with these observations, it has recently been demonstrated, that ALE mediated by CD8 T cells is accompanied by intense infiltrates of monocytes/macrophages and parenchymal microglia activation and triggers the onset of TLE with HS in mice (*29*).

So far, assessment of patients with ALE remains a clinical challenge. Magnetic resonance imaging (MRI) often displays very subtle unilateral or mostly asymmetric bilateral, i.e., lateralized volume and fluid-attenuated inversion recovery (FLAIR) signal increases of the amygdala and anterior hippocampus (*30*–*32*). Electroencephalography (EEG) typically shows unspecific unilateral or mostly asymmetric bilateral, i.e., lateralized anterior temporal epileptic activity and/or slowing (*33*). Moreover, detailed neuropsychological assessment is supposed to distinguish right and left mesial temporal dysfunction, but the spectrum of neuropsychological impairments in ALE is wide (*34*, *35*). Thus, inflammatory CSF changes and the presence of different AABs in serum and CSF serve as the only biomarkers of an autoimmune inflammatory origin (*33*). However, these diagnostic markers remain unremarkable in a considerable number of patients (*35*–*37*). Therefore, more biomarkers aiming to delineate the underlying local immune reaction are urgently needed. Recently, it has been demonstrated that activation of microglia and other innate immune cells in the human brain can be visualized by targeting the 18-kDa translocator protein (TSPO) with second-generation positron emission tomography (PET) tracers such as [^18^F]DPA-714 (*38*). TSPO is located in the mitochondrial outer membrane in particular in microglia and in low levels in neurons and astrocytes in the central nervous system (*39*, *40*), and TSPO reactivity is greatly increased when microglia are activated. Although the exact mechanism is not fully understood, it has been shown that TSPO plays a key role in regulating mitochondrial metabolism. TSPO-KO studies show suppression of mitochondrial oxidative phosphorylation and glycolysis, implying that high expression might on the other hand mirror increased energy demand of the cell upon activation (*41*). First studies on TSPO-PET in TLE hint to increased binding of specific TSPO tracers in current mesial temporal seizure foci but also in the contralateral mesial temporal lobe, suggesting ongoing inflammation largely independent from seizures activity (*42*). This is consistent with the inflammatory changes found in brain tissue specimens of ALE (*10*) and long-standing pharmacoresistant mTLE (*22*–*28*).

Here, we aimed at investigating the potential of TSPO-PET-MRI as a diagnostic marker to assess innate immunity in human CD8 T cell–mediated ALE. Furthermore, back-translation into a newly developed mouse model allows for in-depth analysis of the imaging signal to corroborate the additional diagnostic value of TSPO-PET in ALE. This might pave the way for the clinical use of TSPO-PET-MRI in patients with CD8 T cell–mediated ALE.

## RESULTS

### [^18^F]DPA-714-PET-MRI targeting the TSPO reveals mostly asymmetrically increased binding to mesial temporal lobe structures in patients with ALE

Ten patients (six women and four men; median age, 55 years; range: 22 to 66) suffering from seropositive ALE with AABs against intracellular target antigens or seronegative ALE, both deemed to be meditated by CD8 T cells, underwent combined [^18^F]DPA-714-PET-MRI on clinical grounds and were retrospectively analyzed. Clinical characteristics of the cohort are presented in tables S1 and S2.

**Fig. 1.**
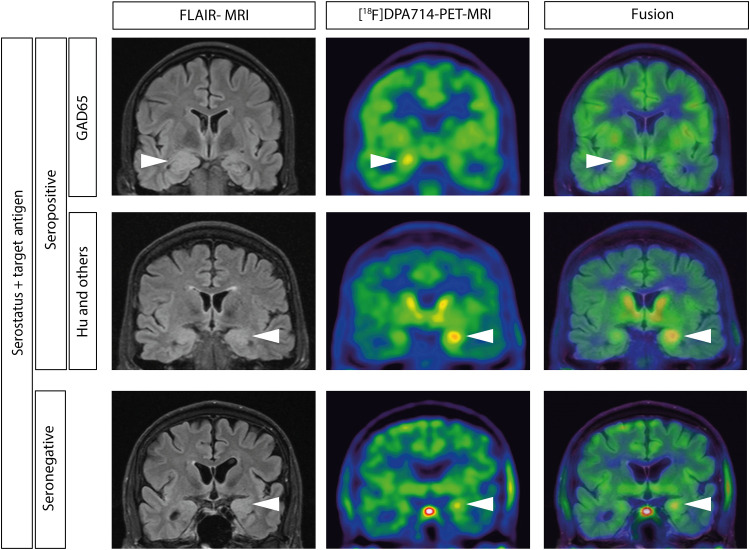
[^18^F]DPA-714 PET-MRI of patients with autoimmune limbic encephalitis. FLAIR-MRI (left), [^18^F]DPA-714 PET (middle), and fused (right) images of three representative patients with seropositive and seronegative ALE [one case of ALE with anti-GAD65 AABs (top), one case of ALE with anti-Hu AABs (and other AABs targeting intracellular neural antigens; center), and one case of ALE without detectable AABs (bottom)]. Focal uptake in amygdala/hippocampus of the affected hemisphere is marked with an arrow.

Images of three representative patients with ALE [one case of ALE with anti-GAD65 AABs, one case of ALE with anti-Hu AABs (and other AABs targeting intracellular neural antigens), and one case of ALE without detectable AABs) are shown in Fig. 1.

All three representative patients displayed unilateral or mostly asymmetrical bilateral FLAIR signal increases corresponding to focal [^18^F]DPA-714 uptake in the amygdala and hippocampus. FLAIR-MRI, [^18^F]DPA-714-PET, and fused images of all other patients demonstrate similar patterns of signal alterations and are shown in fig. S1.

### Quantitative analysis of mesial temporal [^18^F]DPA-714 uptake in patients with ALE reveals lateralization corresponding to mesial temporal FLAIR-MRI alterations and anterior temporal EEG abnormalities but not to mesial temporal volume and memory performance

**Fig. 2.**
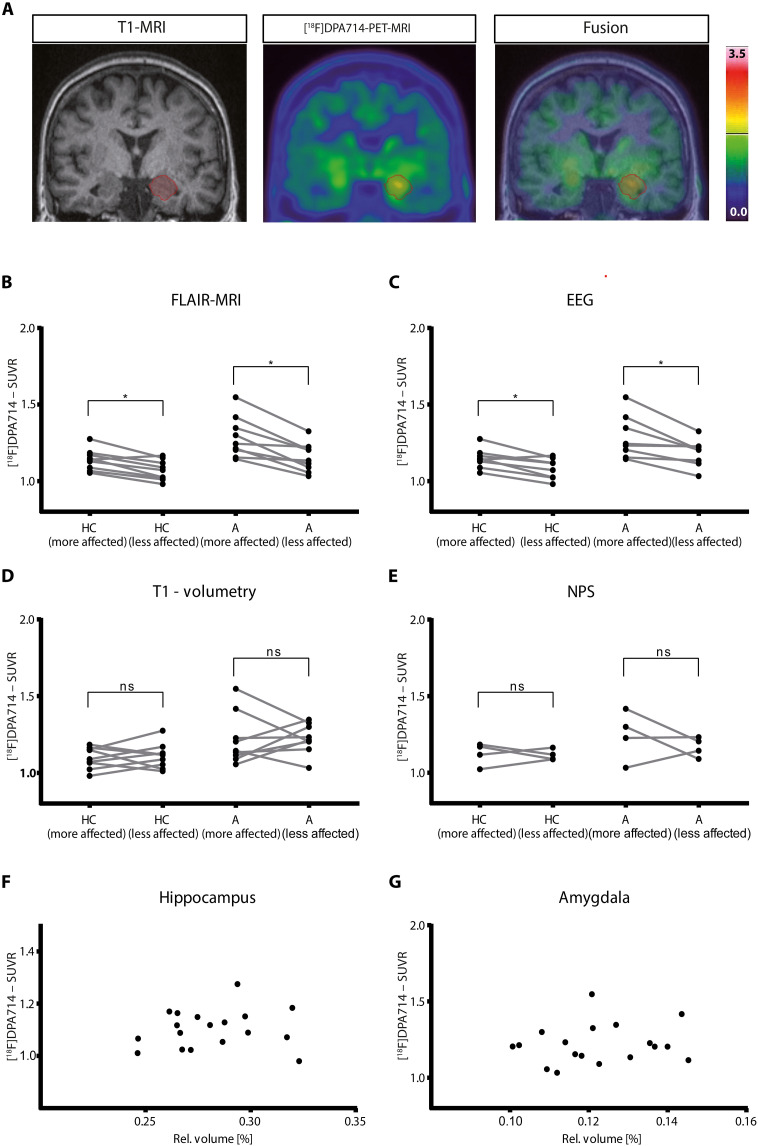
Quantitative analysis of the [^18^F]DPA-714 PET in relation to FLAIR-MRI, EEG, T1-MRI, and neuropsychological functioning of patients with ALE. (**A**) Representative coronal T1-weighted MR (left) and [^18^F]DPA-714-PET (center) image of a patient with ALE with marked left mesial temporal encephalitis (red) and fused image (right). (**B** to **E**) Quantification of the SUVR of the hippocampus (HC) and amygdala (A) of patients with ALE (*n* = 10) according to lateralization of FLAIR signal (B, HC: *P* = 0.008; A: *P* = 0.004), EEG (C, HC: *P* = 0.016; A: *P* = 0.008), T1-volume (D, HC: *P* = 0.570; A: *P* = 0.910), and neuropsychological (NPS) testing (E, HC: *P* = 0.625; A: *P* = 0.625). Cross-correlation between relative MRI–based volume and SUVR of the hippocampus (**F**) and the amygdala (**G**) in patients with ALE (hippocampus, *r*^2^ = 0.13 and *P* = 0.61; amygdala, *r*^2^ = 0.07 and *P* = 0.79). Statistical significance between groups in (A) to (E) was determined with two-tailed Mann-Whitney *U* test or Wilcoxon signed-rank test. Correlation between MRI volume and activity concentration was investigated using Spearman correlation coefficient.

Representative quantitative [^18^F]DPA-714-PET-MRI of an patient with ALE with marked left mesial temporal encephalitis is shown in Fig. 2A. For quantification of the [^18^F]DPA-714 signal, the standardized uptake value ratio (SUVR) with the individual cerebellar gray matter as reference region from 30- to 60-min summation images were used as these values strongly correlated (*r*^2^ = 0.92; *P* < 0.001) to the distribution volume ratio (DVR) from kinetic modeling in a subgroup of six patients suitable for dynamic imaging (fig. S2).

In all patients with ALE, the lateralization of FLAIR-MRI alterations corresponded to that of the standardized [^18^F]DPA-714 tracer uptake (table S1). [^18^F]DPA-714 uptake was significantly higher in hippocampus (*P* = 0.008; more affected, 1.139 ± 0.068, less affected, 1.070 ± 0.066) and amygdala (*P* = 0.004; more affected, 1.285 ± 0.132; less affected, 1.154 ± 0.093) of the hemisphere with predominant mesial temporal FLAIR-MRI alterations (*n* = 9 patients) compared to the corresponding contralateral hemisphere (Fig. 2B).

Moreover, in eight patients with ALE with anterior temporal EEG abnormalities, the lateralization of these alterations also corresponded to that of the standardized [^18^F]DPA-714 tracer uptake (table S1). [^18^F]-DPA-714 uptake was significantly higher in the hippocampus (*P* = 0.016; more affected, 1.148 ± 0.066; less affected, 1.081 ± 0.067) and amygdala (*P* = 0.008; more affected: 1.286 ± 0.141; less affected, 1.183 ± 0.088) of the hemisphere with predominant anterior temporal EEG abnormalities (*n* = 8 patients) compared to the corresponding contralateral hemisphere (Fig. 2C).

[^18^F]DPA-714 uptake was not significantly different in the hippocampus (*P* = 0.570; more affected, 1.097 ± 0069; less affected, 1.109 ± 0.081) and amygdala (*P* = 0.910; more affected, 1.215 ± 0.164; less affected, 1.224 ± 0.096) of the hemisphere with higher T1 volume of hippocampus and amygdala (*n* = 9 patients) compared to the corresponding contralateral hemisphere (Fig. 2D). Consistently, [^18^F]DPA-714 uptake of the respective region did not correlate with relative hippocampal (*r*^2^ = 0.129 and *P* = 0.610; Fig. 2F) and amygdala (*r*^2^ = 0.067 and *P* = 0.791; Fig. 2G) volumes.

Moreover, different degrees of verbal and figural memory impairment assumed to reflect predominantly left and right mesial temporal cognitive functions, respectively, were detected in four patients with ALE. However, in these patients with ALE with significant mesial temporal cognitive dissociation, i.e., functional lateralization (table S1), [^18^F]DPA-714 tracer uptake in the hippocampus (*P* = 0.625; more affected, 1.123 ± 0.073; less affected, 1.115 ± 0.035) and amygdala (*P* = 0.625; more affected, 1.244 ± 0.161; less affected, 1.168 ± 0.064) was not significantly different between the hemisphere with the predominant mesial temporal cognitive dysfunction (*n* = 4 patients) and the corresponding contralateral hemisphere (Fig. 2E).

### TSPO mirrors microglia activation in CD8 T cell–mediated seropositive and seronegative human ALE

**Fig. 3.**
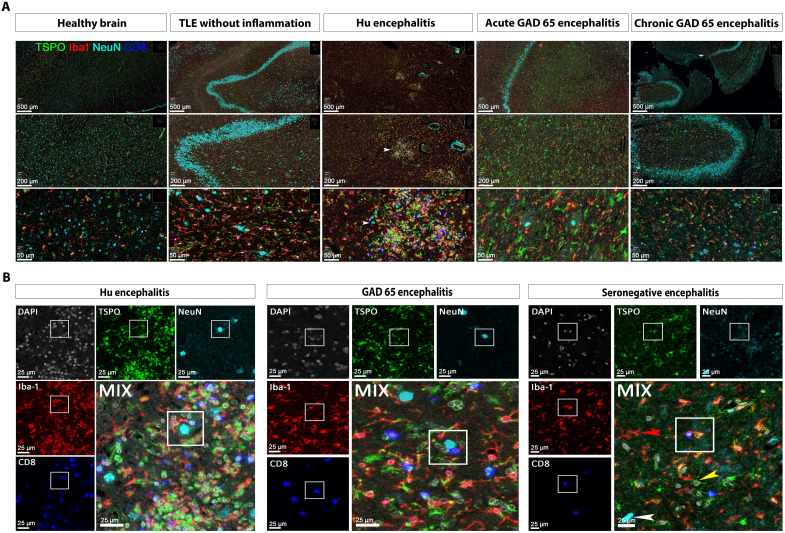
TSPO colocalizes with activated microglia and reactive astrocytes in seropositive and seronegative CD8 T cell–mediated ALE. (**A**) Overview of the TSPO expression pattern in human brain specimen derived from healthy brain, temporal lobe epilepsy without any inflammation, ALE with anti-Hu AABs, and acute and chronic ALE with anti-GAD65 AABs. In healthy brain, TSPO can be found at low levels not only in Iba-1+ microglia but also in other cell types such as endothelial cells, oligodendrocyrtes and astrocytes. Moreover, in TLE without inflammation and without neurodegeneration, Iba-1+ microglial cells are only weakly TSPO reactive. In anti-Hu ALE, TSPO is strongly up-regulated in areas with extensive T cell infiltration, especially mirroring Iba-1^+^ microglia activation. During acute inflammation and neurodegeneration, TSPO reflects mainly reactive gliosis in anti-GAD65 ALE. In the chronic stage of anti-GAD65 where T cell inflammation subsided, TSPO shows moderate activity in microglia and astrocytes. (**B**) Exemplary costaining of TSPO with Iba-1 in CD8 T cell–mediated human seropositive (anti-Hu and anti-GAD65 ALE) and seronegative specimen. In particular, in seropositive anti-Hu ALE and anti-GAD65 ALE, the presence of CD8 T cells is associated with strong TSPO reactivity in surrounding microglia. In seronegative ALE, TSPO^+^ microglia and astrocytes (yellow arrowhead) can be seen together with TSPO-negative microglia (red arrowhead) and TSPO-negative neurons (white arrowhead).

To elucidate the cellular source of [^18^F]DPA-714 uptake in patients with ALE, we performed multicolor immunohistochemistry on human brain specimen. Representative histological staining is shown in Fig. 3. In the healthy brain, TSPO is constitutively present at low levels in various cell types such as endothelial cells and vascular muscle cells, as well as in microglia, astrocytes, and oligodendrocytes but not in neurons (Fig. 3A). In mTLE without inflammation, in areas without neurodegeneration, TSPO expression is minimally enhanced and can be seen in not only microglia but also astrocytes and oligodendrocytes (Fig. 3A). In human specimen from patients suffering from ALE with anti-Hu AABs or from acute ALE with anti-GAD65 AABs, CD8 T cell infiltration was spatially associated with strongly increased TSPO reactivity which overlapped with Iba-1–positive activated microglia and reactive astrocytes (Fig. 3, A and B, and fig. S3). Moreover, increased TSPO reactivity was also found in a case of subacute/chronic ALE with anti-GAD65 AABs. A similar pattern was observed in a patient suffering from seronegative ALE (Fig. 3B). In all cases, most of the NeuN^+^ neurons were TSPO negative. These findings corroborate activated microglia and reactive astrocytes as the main cellular source of [^18^F]DPA-714 uptake spatially associated with CD8 T cell infiltration in human ALE.

### Peripheral immunization induces antigen-specific CD8 T cell–mediated limbic encephalitis in wild-type C57BL/6 mice after AAV-mediated hippocampal neuronal antigen transfer

To corroborate the diagnostic potential of [^18^F]-DPA714-PET-MRI in the context of CD8 T cell–mediated ALE, we back-translated our clinical findings from patients with suspected CD8 T cell–mediated ALE into a novel mouse model of definite CD8 T cell–mediated ALE.

We recently demonstrated that ovalbumin (OVA)–specific T cells can induce ALE and temporal lobe epilepsy when neuronal OVA expression is induced in the CA1 region of the hippocampus of OT-I mice via recombinant adeno-associated virus (rAAV)–vector mediated antigen transfer (*29*). In this context OVA expression resulted in HS within 1 week. To better replicate the human disease course, which is characterized by a subacute inflammation often associated with low numbers of CD8 T cell infiltrates (*10*), we established a novel animal model of mild ALE (termed as “BL6-OVA-LE Model”).

**Fig. 4.**
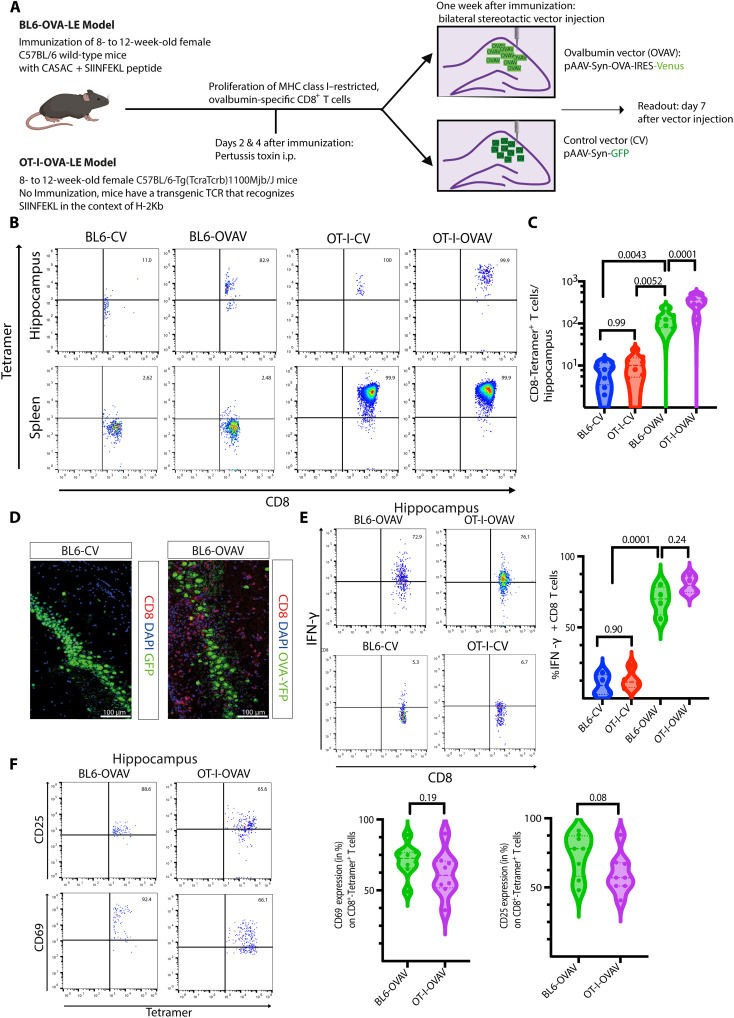
Peripheral immunization induces antigen-specific CD8 T cell–mediated limbic encephalitis in C57BL/6 mice after AAV-mediated hippocampal neuronal antigen transfer. (**A**) C57BL/6 mice were immunized with the SIINFEKL peptide in combination with CASAC and received i.p. 200 ng of PT 24 and 72 hours later. Seven days after the immunization, bilateral stereotactic injections of rAAV vectors into the CA1 region of the hippocampus were performed. Animals received either the OVAV inducing OVA expression together with the yellow-fluorescent protein Venus or a vector inducing emerald green fluorescent protein expression as control (CV) under the neuron-specific human synapsin-1 promoter. To compare our BL6 model to our previously published ALE model, vector injection was also performed in OT-I mice. Experimental readout was performed 1 week after surgery. (**B**) Flow cytometry dot plots representing number and percentage of antigen-specific (Tetramer^+^) CD8 cells in the hippocampus and spleen of immunized C57BL/6 mice and OT-I animals. (**C**) Quantification of antigen-specific T cells in the hippocampus. *n* = 8 per group. BL6-OVAV animals show significantly more antigen-specific T cells in the hippocampus compared to the control groups (BL6-OVAV versus BL6-CV, *P* = 0.0043; BL6-OVAV versus OT-I-CV, *P* = 0.0052) and significantly less compared to OT-I-OVAV mice (*P* = 0.0001). (**D**) OVA expression (green) but not GFP protein (green) expression attracts CD8 T cells (red) into the hippocampus. (**E**) Flow cytometry dot plots show representative intracellular IFN-γ expression (in %) in CD8^+^ T cells. *n* = 8 animals per group; hippocampi of two animals were pooled for analysis. BL6-OVAV and OT-I-OVAV hippocampus-derived antigen-specific cells showed similar (*P* = 0.24) significantly elevated INF-γ expression when compared to the control groups (*P* = 0.0001). (**F**) CD8^+^-Tetramer^+^ T cells derived from the hippocampus of OVAV-injected animals show similar CD25 (*P* = 019) and CD69 (*P* = 0.08) expression. Statistical significance between groups in (C) and (E) was determined with one-way ANOVA and subsequent Tukey post hoc test and in (F), with Student’s *t* test.

In this model, wild-type (WT) C57BL/6 mice were immunized with the combined adjuvant for synergistic activation of CD8^+^ T cellular immunity [CASAC; (*43*)] together with the H-2Kb–restricted peptide (SIINFEKL) of the chicken-derived OVA protein. After 1 week, mice received bilateral stereotactic injection of our previously described rAAV vector plasmids (*29*) encoding either for OVA that is expressed together with the yellow fluorescent protein Venus [OVA vector (OVAV)] or the emerald green fluorescent protein [control vector (CV)] in the CA1 region of the hippocampus (Fig. 4A). Both OVAV and CV drive expression under the control of the neuron-specific human synapsin-1 promoter (hSyn; Fig. 4A). As expected, only OVAV injection resulted in neuronal SIINFEKL presentation via H-2Kb (fig. S4). On days 2 and 4 after immunization, animals received intraperitoneal injection of pertussis toxin (PT) to facilitate T cell migration exclusively into the brain taking advantage of its pleiotropic effect: On the one hand, PT inhibits Gai-coupled receptor–mediated T cell migration (*44*) but on the other, it triggers transient disruption of the blood brain barrier integrity (*45*, *46*) promoting receptor-independent immune cell trafficking into the brain.

Peripheral immunization resulted in about 2.5% antigen-specific (“Tetramer^+^”) T cells in spleen of both, BL6-CV (*n* = 8, 2.32 ± 1.45%) and BL6-OVAV (*n* = 8, 2.2 ± 1.62%) animals (Fig. 4B). When compared to OT-I-OVAV animals, the number of antigen-specific (Tetramer^+^) CD8 T cells in the hippocampus of BL6-OVAV animals was about 2.5 times lower. However, the number of CD8^+^-Tetramer^+^ T cells found in the hippocampus of BL6-OVAV animals was still significantly higher (*P* = 0.0001) compared to the control groups (BL6-CV and OT-I-CV) where almost no antigen-specific CD8 T cells were found (*n* = 8 animals per group; number of CD8^+^-Tetramer^+^ T cells/hippocampus: BL6-CV, 7.75 ± 4.06; BL6-OVAV, 140.5 ± 66.03; OT-I-CV, 10.5 ± 7.45; OT-I-OVAV, 351.9 ± 125; BL6-CV versus OT-I OVAV, *P* = 0.0052; BL6-CV versus OT-I-CV, *P* = 0.99; BL6-CV versus BL6-OVAV, *P* = 0.0043; BL6-OVAV versus OT-I-OVAV, *P* = 0.0001; OT-I-CV versus OT-I-OVAV, *P* = 0.0001; Fig. 4C). Exclusively, in BL6-OVAV animals infiltrations of CD8 T cells were colocated with OVA and SIINFEKL-H-2Kb expression in the neuronal layer, while in the BL6-CV group, only rarely any CD8 T cell was observed (Fig. 4D and fig. S5). Furthermore only CD8 T cells derived from BL6-OVAV and OT-I-OVAV hippocampi showed high levels of interferon-γ (IFN-γ) expression (*n* = 8 animals per group; hippocampi of two animals were pooled for analysis; IFN-γ expression of CD8^+^ T cells: BL6-CV, 9.5 ± 7.3%; BL6-OVAV, 69.0 ± 10.2%; OT-I-CV, 13.3 ± 6.9%; OTI-OVAV, 80.0 ± 6.1%; BL6-CV versus OT-I-CV, *P* = 0.90; BL6-CV versus BL6-OVAV, *P* = 0.0001; BL6-OVAV versus OT-I-OVAV, *P* = 0.24; Fig. 4E). Furthermore antigen-specific CD8 T cells in the hippocampi of BL6-OVAV and OT-I-OVAV animals showed a similar activated-tissue–resident (CD25-CD69^+^) phenotype (*n* = 8; CD25^+^: BL6-OVAV, 73.6 ± 15.7% versus OT-I-OVAV, 60.0 ± 16.2%, *P* = 0.08; CD69^+^: BL6-OVAV, 71.1 ± 11.5% versus OT-I-OVAV, 61.5 ± 16.2%, *P* = 0.19; Fig. 4F).

**Fig. 5.**
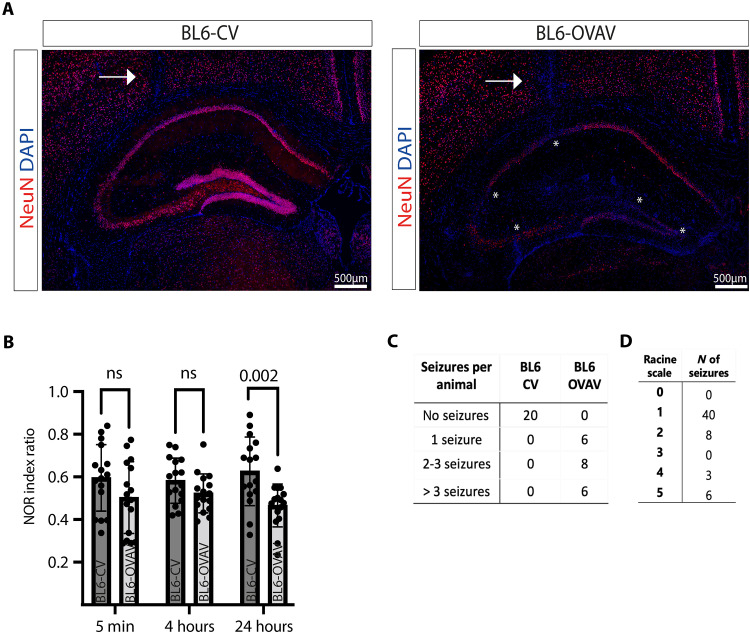
Immunization-induced antigen-specific CD8 T cell–mediated limbic encephalitis in wild-type C57BL/6 mice is associated with neuronal loss, long-term memory impairment, and acute clinical epileptic seizures. (**A**) Representative staining 1 week after vector-based neuronal antigen transfer demonstrates areas with neuron-specific protein NeuN signal loss (*) in the hippocampus of BL6-OVAV animals. In BL6-CV animals, no pathological changes were observed. The white arrow indicates the stereotactic insertion channel. (**B**) NOR displayed significantly impaired long-term (retrieval after 24 hours) spatial memory performance (*P* = 0.001), while short-term and intermediate-term (retrieval after 5 min and 4 hours) spatial memory performance was unaltered in BL6-OVAV (*n* = 20 light gray) compared to BL6-CV (*n* = 20 dark gray) mice within 1 week after vector-based neuronal antigen transfer. Statistical significance was determined by two-way ANOVA with Bonferroni post hoc test. (**C**) Furthermore, all BL6-OVAV animals showed at least one acute clinical epileptic seizure, while in the BL6-CV group, no epileptic seizures were observed. (**D**) Classification of the clinically observed epileptic seizures according to the Racine scale. Most of the animals showed grade I or II epileptic seizures.

One week after vector-based neuronal antigen transfer, areas with neuron-specific protein NeuN signal loss were observed in the hippocampus of BL6-OVAV animals (Fig. 5A, indicated by the white _*_). In contrast, no pathological changes were found in BL6-CV animals (besides the stereotactic insertion channel, indicated by the white arrow).

Furthermore, BL6-OVAV mice displayed significantly impaired spatial memory performance upon delayed (after 24 hours) but not immediate and intermediate (after 5 min and 4 hours) retrieval in the novel object recognition (NOR) test compared to BL6-CV mice, 1 week after vector-based neuronal antigen transfer (*n* = 20 animals per group; 5 min: BL6-CV, 0.6057 ± 0.14; BL6-OVAV, 0.535 ± 0.168; *P* = 0.14; 4 hours: BL6-CV, 0.581 ± 0.111; BL6-OVAV, 0.499 ± 0.119; *P* = 0.07; 24 hours: BL6-CV, 0.613 ± 0.169; BL6-OVAV, 0.467 ± 0.112; *P* = 0.001) (Fig. 5B). These differences were not due to altered anxiety levels in the elevated plus maze (EPM) test (fig. S6), altered motor coordination in the rotarod test (fig. S6), or altered locomotor activity and basal exploratory behavior in the open-field (OF) test (fig. S6), as BL6-OVAV and BL6-CV mice did not show significant differences in these behavioral tests. Moreover, all BL6-OVAV mice displayed one or more acute clinical epileptic seizure within 1 week after vector-based neuronal antigen transfer, while in the BL6-CV group, none of the animals displayed clinically detectable epileptic seizures (Fig. 5C). Most of the seizures (40 of 57) were classified as grade I or II on the modified Racine scale (Fig. 5D).

To conclude, these findings confirm the initiation of a neuron-directed CD8 T cell response in the CA1 region of the hippocampus of SIINFEKL-CASAC–immunized OVAV-injected but not CV-injected BL6 mice within 1 week after vector-based neuronal antigen transfer. Compared to our previously established OT-I OVA LE model, the here presented approach is characterized by lower CD8 T cell numbers resulting in a milder clinical phenotype.

### [^18^F]DPA-714-PET-MRI in mice with CD8 T cell–mediated ALE reveals strong parenchymal innate immune activation

**Fig. 6.**
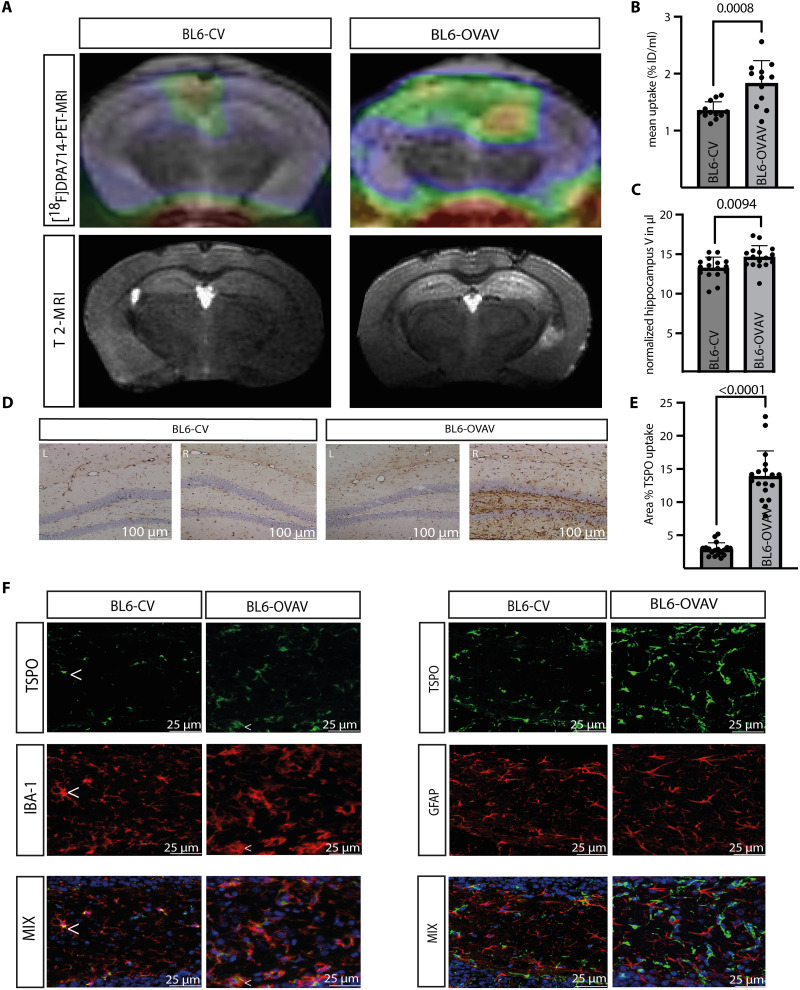
CD8 T cell–mediated limbic encephalitis is associated with hippocampal T2-signal and volume increase as well as significantly increased TSPO-radiotracer uptake. (**A**) Representative [^18^F]DPA-714 PET-MRI images coregistered with the corresponding MRI of a SIINFEKL-CASAC immunized CV (left)– and OVAV (right)–injected BL6 mouse 1 week after vector-based neuronal antigen transfer (top row). T2-weighted images with volume enlargement and generalized edema (bright) as signs of inflammation in the hippocampus in the BL6-OVAV group versus the BL6-CV group. Blood remnants (dark) are detectable on the cortex surface due to bilateral intrahippocampal injection in both experimental groups. (**B**) The mean radiotracer uptake within the right (R) and left (L) hippocampus expressed as percentage of the injected dose per milliliter (%ID/ml) indicated a significant increase in radiotracer uptake in the BL6-OVAV-group compared to the BL6-CV group (*n* = 12, *P* = 0.003). (**C**) Analysis of the MRI-based relative hippocampus volume revealed slight but significant volume enlargement in the BL6-OVAV group compared to the BL6-CV group (*n* = 16, *P* = 0.009). (**D**) Cross-validation of [^18^F]DPA-714 PET imaging by TSPO immunohistochemistry. (**E**) The percentage of TSPO^+^ area was significantly higher in the BL6-OVAV group compared to the BL6-CV-group (*n* = 18, *P* < 0.001). (**F**) TSPO was up-regulated in Iba-1^+^ microglia, while minor expression was detected in GFAP-positive astrocytes in the hippocampus. Nuclei were counterstained with DAPI (blue). Statistical significance between groups was determined by Student’s *t* test or Mann-Whitney *U* test.

MRI 1 week after vector-based neuronal antigen transfer showed bilateral hippocampal T2 signal increases together with significantly increased bilateral hippocampal T2 volumes (*n* = 16 hippocampi of 8 animals per group, *P* = 0.009; BL6-CV: 13.24 ± 1.378 versus BL6-OVAV, 14.63 ± 1.450) in BL6-OVAV mice compared to the BL6-CV group (Fig. 6, A and C). A representative study of [^18^F]DPA-714-PET-MRI performed 1 week after vector-based antigen transfer in BL6-OVAV- and BL6-CV mice is displayed in Fig. 6A. Mean radiotracer uptake (*n* = 8 animals per group; *P* = 0.003; BL6-CV, 1.35 ± 0.15; BL6-OVAV, 1.83 ± 0.40) analysis indicated a significantly increased radiotracer uptake in the BL6-OVAV group compared to the BL6-CV group (Fig. 6B). Radiotracer uptake was cross-validated by TSPO immunoreactivity (Fig. 6, D and E), which showed a significantly increased portion of TSPO^+^ area in the hippocampus of BL6-OVAV animals compared to the BL6-CV group (*n* = 6 animals per group; *P* < 0.001; BL6-CV 2.87 ± 0.99%; BL6-OVAV, 13.89 ± 3.82%). Characterization of the cellular TSPO expression pattern indicated predominant positivity of Iba-1 cells supporting our previous findings in the brain specimen of human CD8 T cell–mediated ALE. Again, minor colocalization between TSPO and GFAP^+^ astrocytes was detected (Fig. 6F).

## DISCUSSION

ALE with acute epileptic seizures is a major cause of chronic temporal lobe epilepsy associated with HS (*2*, *5*). Recently, several studies indicated that CD8 T cells play a key role in the development of ALE in humans and mice (*14*, *29*, *47*–*49*). Unfortunately, diagnostic work-up of patients suspected to suffer from ALE remains a clinical challenge and therefore new biomarkers of mesial temporal lobe autoimmune inflammation are urgently needed. Previous TSPO-PET imaging studies already reported focal TSPO-positive inflammatory tissue reactions in human temporal lobe epilepsy of various etiology, in different animal models of temporal lobe epilepsy, and with different TSPO-PET tracers (*42*, *50*, *51*). To our knowledge, this is the first report on the potential of [^18^F]DPA714-PET-MRI as a diagnostic imaging approach to noninvasively assess innate tissue inflammation in patients suffering from presumably CD8 T cell–mediated ALE. Back-translation into a mouse model of CD8 T cell–mediated ALE in WT C57BL/6 mice allowed for reproduction and in-depth analysis of [^18^F]DPA714-PET signals and their validation down to cellular level in a translational multiscale imaging approach.

We found elevated, mostly asymmetric tracer uptake in the mesial temporal lobe of both hemispheres in presumably CD8 T cell–mediated ALE patients consistent with the notion that ALE usually is a bilateral, albeit often asymmetric, disease (*33*). The lateralization of the [^18^F]DPA-714 signal corresponded very well to that of mesial temporal FLAIR-MRI and anterior temporal EEG alterations but not to neuropsychological assessments of figural and verbal memory functions. Lacking correspondence of memory function to mostly asymmetrical mesial temporal [^18^F]DPA-714 signals in ALE are likely due to the association of visual memory with both mesial temporal lobes (*52*, *53*) and the unavailability of more appropriate memory parameters (*35*, *54*, *55*). Moreover, relative MRI volumes are lacking correlation to differently asymmetrical mesial temporal [^18^F]DPA-714 signals in ALE. This underlines the unique potential of TSPO-PET to directly image key pathophysiological processes in CD8 T cell–mediated ALE and serve as an independent imaging biomarker of parenchymal inflammation and degeneration in addition to AABs and inflammatory CSF changes. However, clinical imaging is lacking a defined experimental environment at defined time points after disease onset, and histopathological correlation is often not feasible in patients with ALE. Moreover, PET imaging of healthy controls, allowing precise age-matched uptake comparison is hampered by unnecessary radiation exposure.

The here described mouse model of antigen-specific CD8 T cell–mediated ALE is an advancement of the recently published model (*29*), which corroborated the key role of CD8 T cells in the pathogenesis of ALE with acute epileptic seizures as precipitating event preceding the development of HS with chronic epilepsy. The former model has several disadvantages: (i) It is based on transgenic mice (OT-I mice) that are characterized by having transgenic inserts for the Tcra-V2 and Tcrb-V5 genes resulting in a homogenous T cell repertoire that exclusively recognizes the MHC class-I–restricted OVA peptide OVA257-264 (SIINFEKL) in the context of H-2Kb (*56*). (ii) OVAV-injection triggers a strong limbic inflammation that was associated with large numbers of CD8 T cell infiltrates and severe destruction of the hippocampal formation already in the early phase and even more pronounced in the later phase of the disease course. In contrast to that, patients suffering from ALE often present with a persisting subtle immune reaction that is associated with subacute to chronic, progressive cognitive impairment, occasional temporal lobe seizures, and persistent mesial temporal FLAIR-MRI (volume and) signal increase without immediate development of severe cognitive deficits, temporal lobe epilepsy, and HS (*57*). In our BL6-OVA LE model we used peripheral immunization of WT C57BL/6 mice to generate antigen-specific CD8 T cells. This resulted in significantly lower numbers of antigen-specific CD8 T cells in the spleen (about 2.5% of OT-I mice) and hippocampus (about 40% of OT-I mice) compared to our previously described OT-I-OVA LE mice. As a consequence, we found a less pronounced immune reaction correlating to less frequent epileptic seizures and less pronounced memory impairment, now only affecting long-term memory abilities. In contrast to that in OVAV-injected OT-I mice both, short- and long-term memory abilities, were strongly affected (*29*). This suggests a more subtle hippocampal inflammatory damage in our model, that appears, however sufficient to elicit epileptic seizures and alter the connectivity between hippocampus and cortex, which is needed for long-term memory formation (*58*). Another clear advantage of this approach is that the immunization model is easily combined with a variety of existing transgenic models based on the C57BL/6 genetic background facilitating future mechanistic investigation of CD8 T cell–driven epileptogenic encephalitis. Furthermore, if a stronger immune response is needed, multiple immunizations could induce larger T cell numbers in the hippocampus. Wells *et al.* (*43*) have demonstrated that three SIINFEKL-CASAC vaccinations in a row lead to about 8.5% of antigen-specific CD8 T cells in the spleen.

Back-translation of the previously established clinical imaging approach into this mouse model of CD8 T cell–mediated ALE revealed bilaterally increased [^18^F]DPA-714 uptake in the hippocampus of OVAV-injected mice compared to controls. In line with clinical imaging results, hippocampal T2 signal (and volume) increases in mice displaying epileptic seizures and memory impairment. These observations are in general agreement with findings in other preclinical (*50*, *51*, *59*) and clinical studies (*42*, *60*, *61*) on seizures and epilepsy.

CD8 T cells recognizing their target antigen expressed by neurons have recently been shown to initiate synaptic pathology and altered neuronal network excitability and degeneration by secretion of IFN-γ. IFN-γ through IFNγR-JAK1/2-STAT1 signaling in neurons causes liberation of CCL2 and subsequent attraction of Iba-1^+^ phagocytes. These phagocytes mediate synaptic loss with subsequently delayed neurodegeneration and are crucial for establishing the clinical symptoms including seizures in mice (*48*, *62*, *63*). Notably, in line with previously reported up-regulation of TSPO in infiltrating immune cells in drug-resistant mTLE (*64*), in the myeloid tumor microenvironment (*65*), and in inflammatory processes as cerebral vasculitis (*66*), Iba-1^+^ phagocytes are the predominant source of TSPO expression in human brain specimen of patients with presumably CD8 T cell–mediated ALE and in our mouse model of definite CD8 T cell–mediated ALE. GFAP^+^ astrocytes express TSPO in particular during reactive gliosis and neurodegeneration (*64*). Thus, translational imaging of TSPO likely enables visualization of a crucial step in the neuronal pathology in humans with CD8 T cell–driven ALE. The initiation of epileptic activity as detected by EEG and the development of tissue edema as detected by FLAIR-MRI are very likely to be downstream effects of the primary T cell–mediated immunopathology.

As in both preclinical and clinical imaging, T2/FLAIR signal alterations occur in the mesial temporal lobe in CD8 T cell–mediated ALE volumetry of FLAIR sequences should be included into future studies. This was not feasible in our pilot study as only two-dimensional (2D) FLAIR sequences were routinely included in the PET-MRI protocol.

There are several potential pitfalls with [^18^F]DPA714-PET-MRI in general and particularly in ALE: (i) A major limitation of [^18^F]DPA-714 PET is reliable quantification and specificity of the signal acquired. The gold standard is kinetic modeling based on dynamic imaging with arterial input function (*42*, *67*, *68*). However, acquisition of arterial input functions is invasive, and potentially harmful for the patients, and therefore, no clinical standard. Alternatively, extracting a reference region by supervised cluster analysis allows for kinetic modeling without the use of arterial input function (*68*). Dynamic imaging over at least 60 min, necessary for kinetic modeling, is often impeded by the clinical status of patients with ALE. Thus, SUVR using cerebellar gray matter as the reference region out of 30- to 60-min postinjection images was established as reliable uptake marker for [^18^F]DPA-714 PET imaging correlating very well with gold-standard kinetic modeling parameters (Fig. 2). (ii) Another more ALE-specific difficulty is potential signal spillover from the choroid plexus that usually shows intense uptake. This might impede reliable signal detection in adjacent regions as the mesial temporal lobe structures. This effect is minimized by applying automated brain segmentation procedures (*42*). (iii) Moreover, inclusion of healthy volunteers in comparison to patients would further strengthen our results. This has to be addressed in a future prospective clinical trial.

To conclude, we provide first translational data on the suitability of [^18^F]DPA-714 PET-MRI as a direct imaging maker of innate inflammation mediated by activated microglia and astrocytes in the context of neuron-directed CD8 T cell–mediated immunity in ALE. These findings underline the potential clinical implication of [^18^F]DPA-714 PET-MRI as a molecular imaging tool that might be used not only as a diagnostic aid in AAB-negative CD8 T cell–mediated ALE but also for patient stratification and response assessment. Future prospective studies are needed to define its role in the clinical workflow. The ability to directly mark predominantly innate inflammation may also apply to a vast variety of other CD8 T cell–mediated brain disorders.

## MATERIALS AND METHODS

### Human patients with ALE

Ten patients with ALE (six women and four men; median age, 55; range, 22 to 66) underwent combined [^18^F]DPA-714-PET-MRI on clinical grounds. All patients were hospitalized in the Section of Epileptology, Department of Neurology, University of Münster, Germany and underwent routine clinical evaluation with either diagnosed or suspected ALE. Diagnosis of ALE was based on current consensus criteria (*33*, *35*). Six patients were classified as seropositive [five patients had AABs against glutamate decarboxylase 65 (GAD65) and one patient had AABs against Hu (and other intracellular neural antigens; table S1)], and four patients were classified as seronegative ALE.

Pro- and retrospective analysis of clinical and paraclinical data, including imaging, were conducted with the ethical standards of the institutional ethics committee (Ethikkommission der Ärztekammer Westfalen Lippe; reference number 2013-350-f-S and 2021-144-f-S) and with the principles of the 1964 Declaration of Helsinki and its later amendments or comparable ethical standards. All patients gave written informed consent on both the study and the genotyping of the Ala^147^Thr polymorphism to characterize individual TSPO binding affinity.

### Neuropsychological assessment in patients with ALE

Neuropsychological assessments were performed after recovery from seizures and were evaluated independently of other findings. To indicate cognitive functioning of the left temporal lobe, we extracted verbal memory scores and to indicate cognitive functioning of the right temporal lobe, we extracted visual memory scores (*69*).

Verbal memory was assessed using the Verbaler Lern- und Merkfähigkeitstest [Verbal Learning and Memory Test (*70*)], which is a word list learning paradigm. Patients are presented with a spoken list of 15 German nouns in five consecutive learning trials. After each trial, patients are asked to recall as many words from the list as possible. The total number of correctly recalled words across the five learning trials is regarded as learning capacity for verbal material.

Memory for visual information was assessed by the Brief Visuospatial Memory Test–Revised (*71*), by the Diagnosticum für Cerebralschädigung II {diagnostic instrument for cerebral damage [DCS-II II; (*72*)]}, or by the Rey Complex Figure Test [RCFT; (*73*)]. A common memory parameter of BVMT-R and DCS-II is learning capacity. Thus, we chose this as shared criterion to relate it to [^18^F]DPA-714 tracer uptake. As the RCFT does not assess learning capacity, we included the parameter immediate memory recall as criterion in this study.

In the RCFT, a complex geometrical figure of 18 details has to be drawn from memory, immediately after a copy trial. Immediate memory recall is evaluated by assigning 0 to 2 points for each remembered detail. In the BVMTR-R, a page with six figures is presented for 10 s. These figures have to be drawn from memory immediately after removal of the presentation page. Each remembered figure can be evaluated by 0 to 2 points. This is repeated over three learning trials. In the end, a sum score is formed over these trials. In the DCS-II, pictures of nine abstract figures each consisting of five straight lines are consecutively presented to the patients one at a time. The patient is asked to recall and reconstruct the nine figures from memory using five wooden sticks. This procedure is repeated until complete reproduction or for up to six learning trials with consecutive recall from memory. Over these trials a sum score is formed of the number of correct reproductions.

Test performances were transformed into standardized *z* values on the basis of age-related norms in the VLMT, BVMT-R, and RCFT and in the DCS-II on the basis of age and education. According to common use in clinical (*74*) and scientific neuropsychological practice (*75*, *76*), a normative *z* value of −1 was regarded as cutoff score for a cognitive deficit. To compare memory functioning of the hemispheres per patient to reveal significant dissociations of functioning, critical differences were computed, correcting for standard measurement error with a 90% confidence interval and correcting for a SE of estimation with an 80% confidence interval with an α value of 0.05. Therefore, we used the retest reliabilities of these tests (*70*, *72*, *73*). Unfortunately, intercorrelations of BVMT-R with VLMT and of RCFT with VLMT are not published, so the intercorrelations of BVMT-R with California Verbal Learning Test and of the RCFT with Rey Auditory Verbal Memory Test were used as estimators (*77*, *78*). The results are shown in table S1.

### Short-term and long-term EEG in patients with ALE

We used the standard 10-20 system of scalp EEG electrode placement. During short-term-EEG recordings, we used additional anterior temporal (T1 and T2) electrodes. For long-term EEG recordings basal temporal electrodes FT9/FT10 and TP9/TP10 were used. The duration of the recordings ranged between 30 min and 9 days depending on the clinical frequency and severity of seizures. For reviewing the EEG records, standard longitudinal bipolar and common average montages were used. The EEG records were rated regarding the presence or absence of anterior temporal interictal epileptic discharges and slowing as well as actual events (max. F7, F8, T1, and T2).

### Routine serum-CSF analysis in patients with ALE

Lumbar puncture was performed under sterile conditions. Samples were processed immediately (within 1 hour) to ensure optimal sample quality. The number of cells was counted in a Fuchs-Rosenthal chamber. Nephelometry was used to measure total protein and immunoglobulin (Ig) levels (IgG, IgA, and IgM). Oligoclonal bands were detected by isoelectric focusing and silver nitrate staining. Protein and Ig levels in serum and CSF were analyzed and a Reiber scheme was created to assess the integrity of the blood-CSF barrier and the quantity of intrathecal Ig synthesis.

Serum and CSF supernatant were analyzed for the presence of IgG autoantibodies against intracellular neural antigens (ANNA1 [Hu], ANNA2 [Ri], ANNA3, PCA1 [Yo], PCA2, Tr/DNER, Ma/Ta, CV2/CRMP5, amphiphysin, SOX1, ZIC4, and GAD65) using a combination of immunoblot and tissue-based assays and against neural surface membrane antigens (NMDAR, AMPAR, GABAaR, GABAbR, GR, LGI1, CASPR2, DPPX, and NRX-3) using a combination of cell-based and tissue-based assays according to manufacturer’s instructions (EUROIMMUN, Lübeck, Germany).

CSF samples were negative for viral, fungal, and bacterial pathogens, and an extensive serum panel for rheumatological-vasculitic disorders (ANA, ENA, ANCA, RF, ds-DNA-abs, ACE, and phospholipid-abs) was negative.

### [^18^F]DPA-714 PET-MRI in patients with ALE

Imaging data were acquired on a 3-T Siemens PET-MRI system (mMR; Siemens Healthcare GmbH, Erlangen, Germany). Dynamic PET data were acquired in list mode after bolus injection of 247 ± 19 MBq [^18^F]DPA-714. On the basis of anticipated kinetics of [^18^F]DPA-714, list mode data were obtained for 60 min from time of injection. Patients not suitable for dynamic imaging (*n* = 4) underwent static PET 30 to 60 min after injection. Non–contrast-enhanced MRI sequences, including high-resolution isotropic (1 mm) 3D structural T1-weighted, axial T2-weighted sequences, and axial and coronal FLAIR sequences were obtained. In one patient (#5), no T1 3D sequence was available for retrospective analysis.

### DNA extraction and TSPO polymorphism genotyping in patients with ALE

Patients were tested for single-nucleotide polymorphism c.439A > G (rs6971, p.Thr^147^Ala) by direct Sanger sequencing, known to affect the binding affinity of second-generation TSPO-PET-tracers. Genomic testing was performed at the Institute of Human Genetics, University of Münster, Münster, Germany as described previously (*66*).

### Analysis of human [^18^F]DPA-714 PET-MRI

All 3D T1-weighted MR images were corrected for contrast and intensity inhomogeneities to reduce segmentation errors. Volumes of hippocampus and amygdala and intracranial volume (ICV) were obtained with FreeSurfer software, version 7.2, which is fully documented and freely available for download online (http://surfer.nmr.mgh.harvard.edu). Absolute hippocampus and absolute amygdala volume were corrected for ICV to account for individual variations in head size [see (*79*) for exact formula].

PET and MRI images were acquired simultaneously, and therefore, the images were already coregistered. Despite this, the registration was checked manually to ensure that there was no coregistration errors due to patient movement.

For the images where dynamic data were available (*n* = 6), parametric maps of DVR and binding potential (BPnd = DVR-1) were estimated using a voxel wise Logan plot (code for estimation was downloaded from the Turku PET Centre: https://gitlab.utu.fi/vesoik/tpcclib) with a reference region extracted using a supervised cluster analysis method (*68*).

Segmented T1-weighted MR images were used to extract regional time activity curves from the dynamic images (where available) and the summed activity over 30 to 60 min for all images. For all images, the SUVR using the cerebellar gray matter as reference region was calculated.

Regional values of DVR, BPnd, and SUVR were extracted from the images using the segmented MRI values. Regions of interest used were hippocampus and amygdala. A correlation analysis was performed between the DVR and the SUVR where dynamic images were available (*n* = 6) (fig. S2). This was done to see whether the SUVR using cerebellar gray matter as the reference region is appropriate as a binding parameter in this pathology.

### Immunofluorescence staining in human ALE brain samples

Available surgical specimens from patients with ALE were stained with anti-TSPO antibodies (Abcam ab109497; 1:3000). In short, sequential 4-μm sections of paraffin-embedded hippocampal specimens were deparaffinized and antigen retrieval was performed by steaming in a conventional household food steamer with in tris buffer (0.01, pH 8.5) with EDTA (0.05 M) for 30 min. Primary antibody was applied over night at 4°C. Incubation with primary antibodies was followed by a biotinylated donkey–anti-rabbit antibody (Jackson #711-065-152), avidine-peroxidase (Jackson #016-030-084) and DAB development.

### Multiplex immunofluorescent labeling

We performed multiplex immunofluorescent labeling for neurons (NeuN; Chemicon #MAB377; 1:2500), T cell subset CD8 (Dako #M7103; 1:500), microglia (Iba-1; Wako #019-19741; 1:10.000), and TSPO (Abcam ab109497; 1:10.000) by using the Akoya Fluorescent Multiplex kit according to the manufacturer’s protocol. In brief, sections were steamed in antigen retrieval buffer pH 6.0 (Akoya Biosciences, Marlborough, MA, USA, AR6) for 60 min in a household food steamer (Braun) followed by a 10-min blocking step with Opal Antibody Diluent/Block solution (Akoya). The incubation time for the primary antibodies was 2 hours at room temperature or overnight at 4°C. Subsequently, the sections were rinsed in tris-buffered saline with Tween 20. Opal polymer horseradish peroxidase (HRP) Ms. + Rb (PerkinElmer Boston, USA) was applied for 10 min at room temperature. Subsequently, the sections were incubated with one of the fluorophores (Opal 480, Opal 570, Opal 570, Opal 690, and Opal 780). Before introducing the next primary antibody, the sections were fixed with 4% paraformaldehyde for 10 min at room temperature followed by another antigen retrieval step using AR6 for 30 min.

### Radiochemistry for preclinical and clinical [^18^F]DPA-714-PET

[^18^F]DPA-714 was prepared automatically in a GE TRACERlab MX module in accordance to a published procedure (*80*) and as described in detail previously (*66*).

### Mice

All animal work was performed in accordance with the 2010/63/EU of the European Parliament and Council of 22 September 2010 and has been approved by local authorities (Landesamt für Natur, Umwelt und Verbraucherschutz Nordrhein-Westfalen; approval IDs: 84-02.04.2014.A109 and 81-02.04.2019A441). All efforts were made to minimize the number of animals used and to avoid their stress and suffering by strictly following the ARRIVE guidelines (*21*). Eight- to 12-week-old female C57BL/6 mice and OT-I mice were used for all experiments. Mice were individually caged, kept in a 12-hour light/dark cycle, and food and water were available ad libitum.

### Immunization of mice with the H2Kb-restricted SIINFEKL peptide together with the CASAC

C57BL/6 mice were immunized intraperitoneally (i.p.) with CASAC (*43*) to the H2Kb-restricted SIINFEKL peptide containing the following reagents and their amount per mouse in double-distilled water: antigen peptide, SIINFEKL (100 μg; AS-60193-1 Eurogentec); stimulating antibody, anti-CD40 (clone 3/23, 25 μg; MCA1143 Bio-Rad); T helper 1 cell cytokine, IFN-γ (100 ng; Peprotech); Toll-like receptor 4 (TLR4) agonist emulsion, monophosphoryl lipid A + trehalose 6,6-dimycolate emulsion (Sigma Adjuvant System, used as per manufacturer’s instructions; Sigma-Aldrich); and TLR9 agonist, CpG 1826 (25 μg; Invivogen). Final vaccine volumes were 200 μl. Vaccines are warmed to 37°C and shaken vigorously before injection. All mice are injected i.p. with PT (400 ng; List Biological Laboratories) 24 and 72 hours after immunization.

### rAAV vector construction and production

rAAV vector plasmids encoding either the yellow fluorescent Venus sequence together with OVA (pAAV-hSyn-OVA-IRES-Venus, OVAV) or the emerald green fluorescent protein alone (EmGFP; pAAV-hSyn-EmGFP, CV) under the control of the neuron-specific hSyn were constructed as described previously (*29*, *81*).

### rAAV vector–based neuronal antigen transfer

One week after immunization, C57BL/6 mice were anesthetized i.p. with ketamin/xylazine [100/10 mg/kg body weight (BW) in 10 μl/g BW phosphate-buffered saline (PBS) i.p.] and placed into a stereotactic frame. Intracerebral injection of either OVAV or CV particles in both CA1 hippocampal regions was performed stereotactically at the coordinates −2-mm posterior, −2-mm lateral, and 1.7-mm ventral relative to bregma (*23*). The skin was incised, and holes of the size of the injection needle were drilled into the skull, and 1 μl of viral suspension containing ∼10^8^ transducing units per CA1 region was injected with a 10-μl Hamilton syringe at a rate of 100 nl/min using a microprocessor controlled mini-pump (World Precision Instruments). After injection, the needle was left in place for 5 min before withdrawal, and the skin was closed by stitching. The mice were allowed to recover from anesthesia, and postoperative analgesia was performed using subcutaneous (s.c.) application of carprofen (4.0 to 5.0 mg/kg BW in 10 μl/g BW PBS s.c.).

### Flow cytometry of hippocampal leukocytes

One week after rAAV vector–based neuronal antigen transfer, flow cytometric analysis of hippocampal leukocytes was performed as follows. For preparation of hippocampus-invading leukocytes, mice were perfused transcardially with PBS to diminish contamination by leukocytes located within the blood vessels. To take into account not only hippocampal but also hippocampus-surrounding infiltrates, we avoided to extract the hippocampus only. Therefore, the brain was roughly reduced to a tissue block by dissociating the frontal brain part, cerebellum, and brain stem. Then, the tissue block was dissociated mechanically followed by enzymatic digestion with collagenase CLS2 (Worthington) and deoxyribonuclease (Sigma-Aldrich) for 45 min at 37°C. After two washing steps, the cell suspension was transferred to a 30/50% Percoll (Amersham) density gradient. After centrifugation (2500 rpm, 30 min, 20°C) mononuclear cells were isolated from the interface of the gradient and counted by a Casy Model TT cell counter (Innovatis).

For Fc-receptor blocking, cells were incubated in fluorescence-activated cell sorting (FACS) buffer (eBioscience) for 20 min at 4°C with a CD16/32 antibody (BioLegend, clone 93, 1:50). For cell surface staining, cells were washed with FACS buffer and stained with appropriate antibodies in the same buffer for 30 min at room temperature in the dark. Cells were centrifuged and stored in PBS with 0.5% formaldehyde (Merck) at 4°C protected from light for a minimum of 1 hour (maximum 24 hours) before analysis by flow cytometry.

The last washing step was performed with FACS buffer, followed by resuspending in FACS buffer with calibrate beads (BD Biosciences) and flow cytometry. Cells were analyzed on a Gallios Flow Cytometer (Beckman Coulter). Antibody concentrations were carefully titrated before experiments. The following primary anti-mouse antibodies were used for flow cytometry together with respective isotype controls: CD3 PerCP/Cy5.5 (BioLegend, 17A2) 1:100, CD4 Brilliant Violet 510, (BioLegend, GK1.5), CD8 FITC (MBL, KT15) 1:50, iTAg Tetramer/APC–H-2Kb OVA (SIINFEKL) (MBL) 1:20, CD25 PE/Cy7 (BioLegend, PC61) 1:100, CD69 eFluor 450 (eBioscience, H1.2F3) 1:5, 1:100, and IFN-γ Brilliant Violet 421 (BioLegend, XMG1.2) 1:50.

Intracellular staining was performed with BD Cytofix/Cytoperm Plus (with GolgiPlug; BD Pharmingen) according to the manufacturer’s instructions. For the measurement of IFN-γ–producing CD8 T cells, 2 × 10^6^ splenocytes/ml or a single-cell suspension prepared from hippocampi was incubated with 5 μM OVA_257–264_ (SIINFEKL) peptide and GolgiPlug (1 μl/ml) for 6 hours. Live/dead staining was performed with eBioscience Fixable Viability Dye eFluor 780 (Thermo Fisher Scientific, 65-0865-14), 1:1000. The exact gating strategy to identify CD8 T cells is demonstrated in fig. S7.

### Immunohistochemistry in the mouse model

Immunohistochemistry was performed 1 week after bilateral intrahippocampal rAAV vector injection. Briefly, mice were deeply anesthetized using ketamine/xylazine (100/10 mg/kg BW in 10 μl/g BW PBS i.p.) and then transcardially perfused as described using phosphate saline buffer (PBS). Afterward, the brains were quickly removed, embedded in cryoprotective compound (TissuTeK, Science Service), and frozen using liquid nitrogen. Coronal cryo-sections (10 μm thickness) were cut using a cryotome (Leica) and positioned on a glass slide (three per slide) and conserved at −20°C. Slices were fixed in a solution containing 4% paraformaldehyde (PFA) for 10 min and then washed with PBS. To avoid false-positive results, slices were incubated for overnight at 4°C with a blocking solution composed of a blocking reagent (11096176001, Roche) and 0.03% Triton X-100. After blocking, the slices were incubated with the following primary antibodies: CD8 (Alexa Fluor 594 mouse anti-mouse CD8 antibody, clone 53-6.7 BioLegend, 1:250; and for supplement 7, mouse–anti-mouse–Alexa Fluor 488, clone 53-6.7 BioLegend, 1:250), a marker of CD8 T cells; NeuN (Alexa Fluor 647 rabbit anti-mouse NeuN antibody, ab190565, Abcam 1:500) a marker for neuronal somata; SIINFEKL:H-2Kb (APC mouse anti-mouse SIINFEKL:H-2Kb antibody, clone 25-D1.16, BioLegend, 1:100) a marker of the SIINFEKL:H-2Kb complex; and anti-GFP (rabbit polyclonal anti-GFP antibody ab290, Abcam), to enhance the signal of the GFP protein expressed by the CV or the yellow fluorescent protein expressed by the OVAV.

Antibodies were diluted in a cold solution in the previously described blocking reagent. Overnight incubation was performed. After washing with PBS, the mounting medium Fluoromount-G containing 4′,6′-diamidino-2-phenylindole (DAPI; Invitrogen by Thermo Fisher Scientific) was used as marker for cell nuclei.

Images were acquired using a ZEISS Axio Imager Z1 with ApoTome. Images of slices containing the hippocampus were collected from both hemispheres. For overview images, the KEYENCE BZ-9000E microscope was used.

### MRI in the mouse model

MRI was performed 1 week after rAAV vector–based neuronal antigen transfer using a 9.4-T small-animal MRI scanner (BioSpec 94/20; gradient strength, 720 mT/m) equipped with a helium-cooled CryoProbe (Bruker BioSpin MRI GmbH). T2-weighted 3D scans were acquired. Geometry parameters were a field of view of 14 mm × 14 mm × 14 mm and an acquisition matrix of 160 × 160 × 96, resulting in an in-plane resolution of 70 × 70 μm and a slice thickness of 300 μm. Scan time per animal was 6 min and 43 s, repetition time of 5600 ms, and echo time of 60 ms. Other parameters were Rapid Acquisition with Refocusing Echo (RARE) factor 16, six averages, and fat suppression. Scanning was performed under inhalation anesthesia using 1.5% isoflurane (1 liter/min in O_2_/compressed air, 20/80).

### MRI image processing in the mouse model

The hippocampal volume was calculated by analyzing T2-weighted sequences with Amira Software (Thermo Fisher Scientific). For better comparison, the hippocampus volume was normalized by using the formula: raw hippocampal volume / raw brain volume × mean of all brain volumes.

### Behavioral assessments in the mouse model

One week after rAAV vector–based neuronal antigen transfer, all mice underwent a series of behavioral tests to evaluate locomotor activity, motor coordination, anxiety level, and memory.

The OF [Ethovision, Noldus; (*82*, *83*)] test was performed to evaluate locomotor activity and basal exploratory behavior of mice. The time spent grooming, as well as the frequency of rearings (animal put its weight on his hind legs, raises its forelimbs from the ground, and extends its head upward) were measured manually. Animals were tested in the OF arena (35 cm by 40 cm by 40 cm). The traveled distance and velocity as well as the time spent in the periphery were taken as a readout.

The EPM [Ethovision, Noldus; (*28*)] was used to assess anxiety-like behavior of mice as described previously (*29*). The EPM system is elevated from the floor (50 cm) and consists of two closed and two open arms, which the animal is allowed to explore for 5 min. Each group underwent the test once, and the time spent in closed and open arms was taken as readout.

The static rotarod test (*29*) was used to assess motor coordination. In this test, the mouse is placed on a horizontal rod, which rotates at 10 rotations/min about its long axis. At least two flanges prevent the mouse from leaving the rod. They are typically 30 cm in diameter (but this can vary) and are separated at 6 cm. The task of the mouse is to walk forward on the rotating rod (rotarod) without falling off. The mouse must walk forward for a trial to be counted. Holding on to the rod but not walking results in “cartwheeling” and invalidates the trial. The drop latency to fall off time was measured up to a maximum total time of 5 min after a habituation phase to the rod. Each animal underwent five trials, if the animal has reached 5 min on the road, the trial was stopped.

The NOR [Ethovision, Noldus; (*30*)] test was performed to evaluate cognitive and memory abilities of the animals (all time points). The same arena was used as for the OF, since the animals were already familiar with it. The test consists of a habituation phase, where animals are allowed to explore two identical objects for 10 min and three retrieval phases performed at different time intervals after habituation to evaluate short-term nonhippocampal-related (5 min), short-term hippocampal-related (4 hours), and long-term memory (24 hours) abilities (*29*, *84*, *85*). For each retrieval session one of the old objects was substituted by a novel one (chess pieces were used for all tests) at a novel position. The time spent exploring the novel and the old object were used to calculate a NOR index (*30*) as: (time novel)/(time novel + time old). An index >0.5 indicates that animals explore the novel object more than the old one, suggesting proper memory skills; an index = 0.5 indicates that animals explore both novel and old object, suggesting their inability to distinguish the novel from the old one (*85*).

### Seizure monitoring and seizure classification in the novel mouse model

In addition to normal routine handling and experimentation (behavioral assessments), all mice were monitored 2 hours per day for the occurrence of epileptic seizures throughout the experiment by two observers blinded for the experimental groups. All spontaneous focal and generalized seizures were classified as described (*86*), with stage I (immobility and rigid posture), stage II (repetitive movements and head bobbing), stage III (severe seizures with rearing without falling), and stage IV (severe seizures with rearing and falling/loss of righting ability). Tonic-clonic seizures were classified as stage V.

### Preclinical [^18^F]DPA-714 PET-CT/-MRI imaging in the mouse model

[^18^F]DPA-714 PET imaging was carried out with a high-resolution small-animal PET scanner (32 module quadHIDAC, Oxford Positron Systems Ltd.) (*87*). Mice received 12.3 ± 0.6 MBq of [^18^F]DPA-714 tracer via tail vein under anesthesia. After i.v. injection, mice were kept under anesthesia for 45 min and scanned from 45 to 65 min after injection. PET data were reconstructed using one-pass list mode expectation maximization algorithm with resolution recovery (*87*, *88*). Data were corrected for activity decay but not for partial volume effects. After the PET scan, the animal bed was immediately transferred into a computed tomography (CT) scanner (Inveon, Siemens Medical Solutions, Knoxville, TN, USA).

Three sieve spheres (Acros Organics, Geel, Belgium) were used as landmarks to coregister PET and CT images. They were rinsed in radiotracer solution and fixed at the extremities of the bed, visible by PET and CT imaging.

MR imaging after PET scan was conducted to localize the hippocampi and detect any sign of tissue damage. MR imaging was carried out with a 1-T nanoScan PET/MRI scanner equipped with a MH20 coil (Mediso Medical Imaging Systems). A T2 FSE 2D axial sequence was used with the following parameters: repetition time, 5253 ms; effective echo time, 44.25 ms; number of slices, 24; slice thickness, 0.80 mm with a gap of 0.1 mm; and rare factor, 30.

### Preclinical image analysis of [^18^F]DPA-714 PET-CT/-MRI in the mouse model

The full in vivo imaging dataset was analyzed using the in-house–developed software MEDgical dedicated to the analysis of multidimensional, multiscale biomedical image data, as previously described (*89*, *90*). PET-CT images were manually coregistered with the T2-weighted MR images following the bone structure and ventricles, overlaid by an atlas. Right and left atlas-based hippocampi were manually adjusted to the T2-weighted MR images. Mean and maximal radiotracer uptakes were assessed within the hippocampal region and data were reported in percentage of injected dose per milliliter in both regions.

### Immunohistochemistry in mice undergoing [^18^F]DPA-714 PET

Mouse brains were fixed in 4% PFA, embedded in paraffin, and cut in 5-μm coronal sections. After deparaffinization, brain slices were boiled in citrate buffer (pH = 6, 15 min) and washed three times in deionized water and PBS.

For immunohistochemistry, the sections were incubated in hydrogen peroxide for 10 min to block endogenous peroxidase activity (ab64218, Abcam, Cambridge, UK). Bain sections were incubated with the primary antibody overnight at 4°C: anti-PBR (anti-TSPO) (rabbit, 1:250, ab109497, Abcam, Cambridge, UK). Then, slices were incubated with biotinylated goat anti-rabbit (1:800 in blocking buffer, 45 min, B21078, Life Technologies, Darmstadt, Germany) followed by HRP-streptavidin incubation (1:600 in PBS, 30 min, K1016, DAKO, Hamburg, Germany). The staining was visualized after incubation with 3,3-diaminobenzidine (D-5637, Sigma-Aldrich, St. Louis, USA) for 3 min. Sections were counterstained with hematoxylin, dehydrated, and mounted using Entellan (Merck, Darmstadt, Germany).

### Immunofluorescence in mice undergoing [^18^F]DPA-714 PET

For immunofluorescence, the sections were processed using the following primary antibodies: red fluorochrome (635)–conjugated anti-ionized calcium binding adapter molecule 1 (anti-Iba-1) (rabbit, 1:500, 013-26471, Wako Chemicals USA Inc., Richmond, VA, USA), anti-PBR (anti-TSPO) (rabbit, 1:250, ab109497, Abcam, Cambridge, UK), recombinant Alexa Fluor 488 anti-PBR [EPR5384] (anti-TSPO) (rabbit, 1:250, ab199779, Abcam, Cambridge, UK), and anti–glial fibrillary acidic protein (anti-GFAP) (chicken, 1:500, ab4675, Abcam, Cambridge, UK). Sections were incubated overnight at 4°C with the primary antibody, followed by incubation for 45 min at room temperature with the corresponding secondary antibody. Sections were counterstained with DAPI (Invitrogen) to visualize the cell nuclei. Images were acquired using a confocal laser microscope. Images were processed with Fiji software.

### Statistical analysis and data statement

For analysis of clinical and preclinical imaging data, comparison between two nonnormally distributed and nonmatched groups was carried out with two-tailed Mann-Whitney *U* test. Matched comparisons without normal distribution were carried out with Wilcoxon signed-rank test. Student’s *t* tests, two-tailed Mann-Whitney *U* test, and one-way and two-way analysis of variance (ANOVA) followed by Tukey or Bonferroni’s multiple comparison tests were used to evaluate the statistical significance of the results in the development of the mouse model. Tests were used after testing for normal distribution (Shapiro-Wilk) and equal variance (Brown-Forsythe). Correlation between MRI volume and activity concentration was investigated using Spearman correlation coefficient. Values were considered significant at *P* < 0.05. All results are plotted as the mean ± S.D.

## Supplementary Materials

### This PDF file includes:

Figs. S1 to S7 Tables S1 and S2 Legend for auxiliary Excel file with raw data

### Other Supplementary Material for this manuscript includes the following:

Auxiliary Excel file with Raw Data
