# Supplementary material for: Translational imaging of TSPO reveals pronounced innate inflammation in human and murine CD8 T cell–mediated limbic encephalitis
Source: Sci Adv. 2023 Jun 9;9(23):eabq7595. doi: 10.1126/sciadv.abq7595 (PMC10256169; doi:10.1126/sciadv.abq7595)
Supplement: Supplementary file 1 — Figs. S1 to S7 Tables S1 and S2 Legend for auxiliary Excel file with raw data [file sciadv.abq7595_sm.pdf]

Science Advances
AAAS

Supplementary Materials for

Translational imaging of TSPO reveals pronounced innate inflammation in human and murine CD8 T cell-mediated limbic encephalitis

Marco Gallus *et al.*

Corresponding author: Nico Melzer, nico.melzer@med.uni-duesseldorf.de

Sci. Adv. **9**, eabq7595 (2023)
DOI: 10.1126/sciadv.abq7595

The PDF file includes:

Figs. S1 to S7
Tables S1 and S2
Legend for auxiliary excel file with raw data

Other Supplementary Material for this manuscript includes the following:

Auxiliary Excel file with Raw Data

Supplement

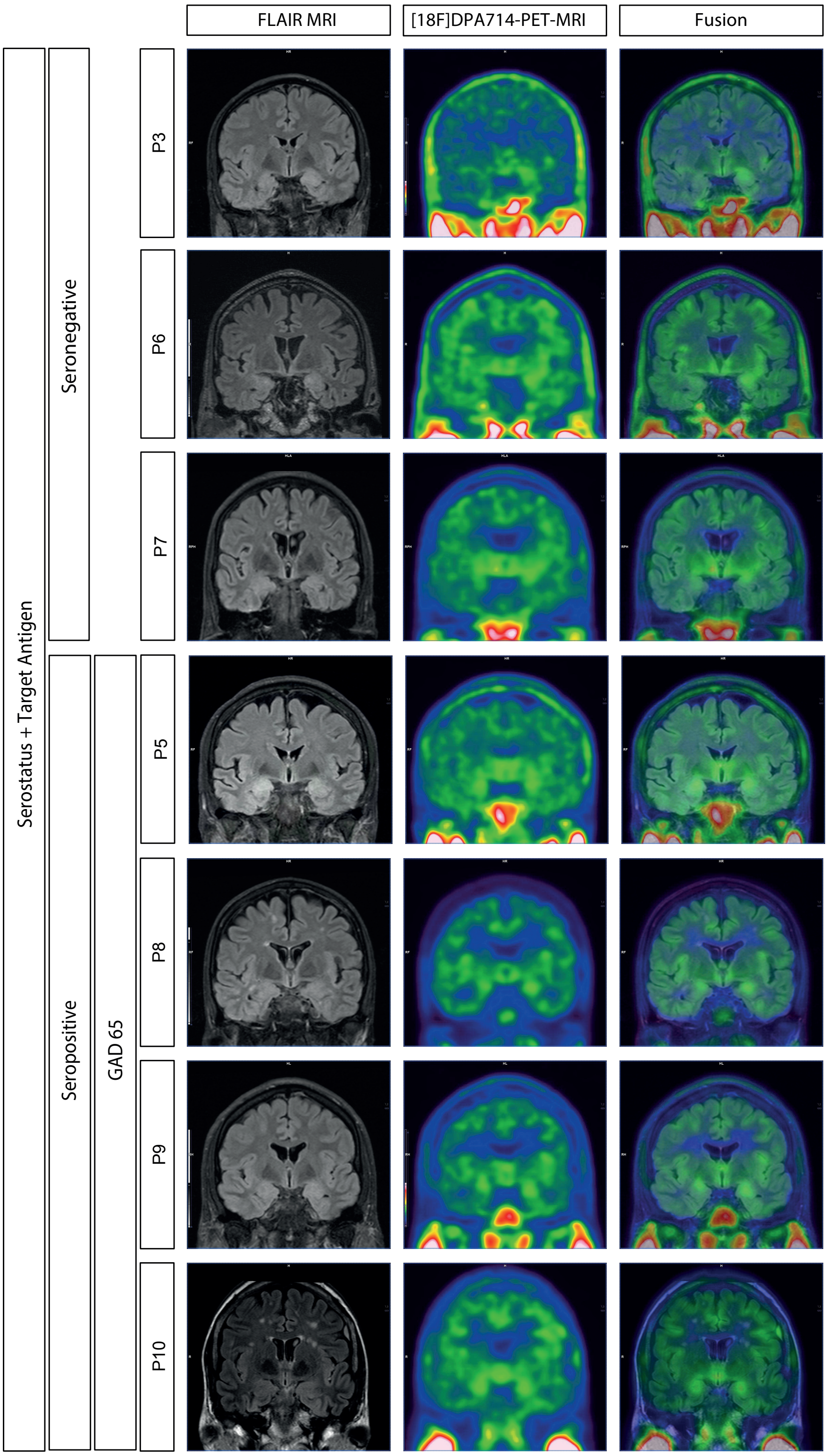

Supplement Figure 1: [^{18}F]DPA-714-PET-MRI in patients with autoimmune limbic encephalitis. FLAIR-MRI (left), [^{18}F]DPA-714 PET (middle) and fused (right) images of all patients not displayed in Figure 1 with bilateral/unilateral dominant tracer uptake.

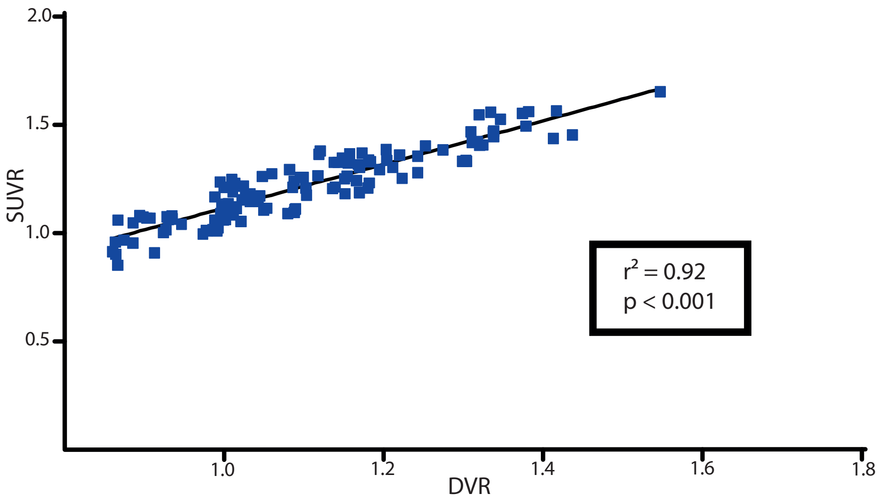

Supplement Figure 2: Correlation of kinetic modelling-derived and quantitative relative uptake parameter of [^{18}F]DPA-714-PET in patients with autoimmune limbic encephalitis.

A correlation analysis was performed between the kinetic modelling-derived distribution volume ratio (DVR) and the relative standard uptake value (SUVR) in patients with ALE in whom dynamic images were available (N = 6 patients). This was performed in order to see whether the SUVR using cerebellar grey matter as the reference region is appropriate as a binding parameter in this pathology. Correlation between DVR and SVR was very strong (r>0.9) and thus regarded as sufficient to use SUVR with cerebellar grey matter as the reference region for further analysis.

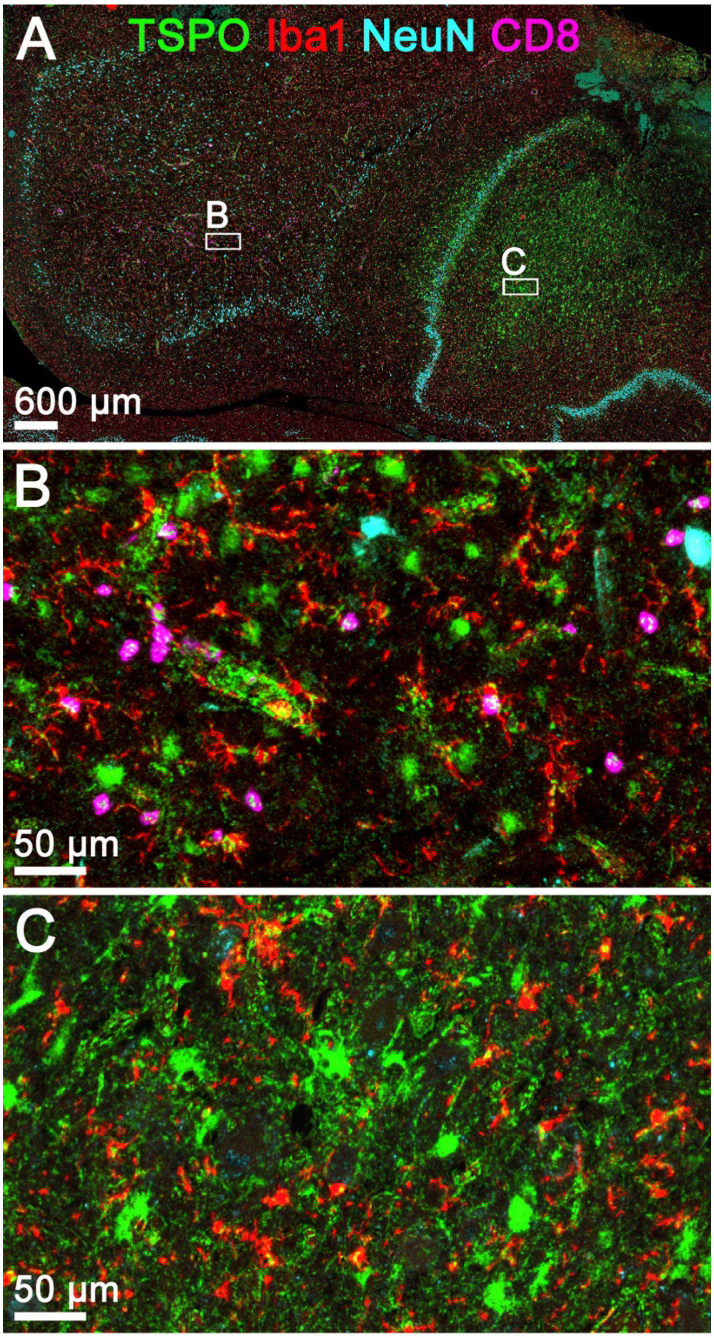

Supplement Figure 3: TSPO in acute GAD LE Multiplex staining for TSPO (green), Iba1 (red), NeuN (Cyan) and CD8 (magenta). (A) Overview of part of the hippocampus. Two CA4 regions surrounded by the dentate gyrus are indicated by rectangles B and C. **(B)** This region shows strong inflammation by CD8 T cells while TSPO can be seen upregulated in Iba1^{+} microglia and astrocytes. Multiple NeuN^{+} neurons are present. **(C)** This region shows an even more intense TSPO staining, especially in swollen astrocytes indicating reactive gliosis. CD8 T cells are rare while NeuN^{+} neurons are lost from this area.

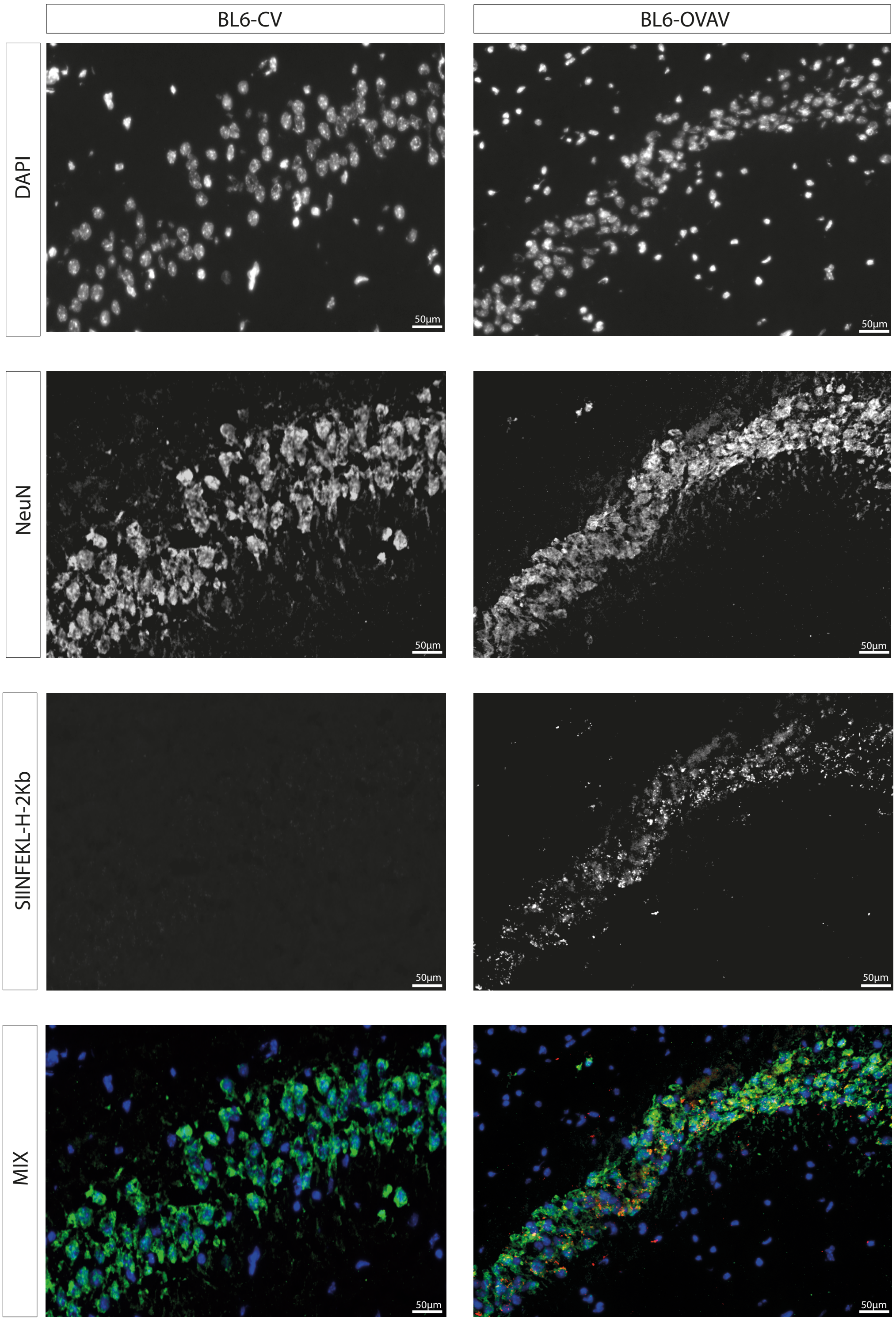

Supplement Figure 4: SIINFEKL-H-2Kb expression in the hippocampus of BL6-OVAV vs. BL6-CV mice. NeuN (green), DAPI (blue), SIINFEKL-H-2Kb (red). SIINFEKL-H-2Kb expression was exclusively found in the hippocampus of BL6-OVAV mice.

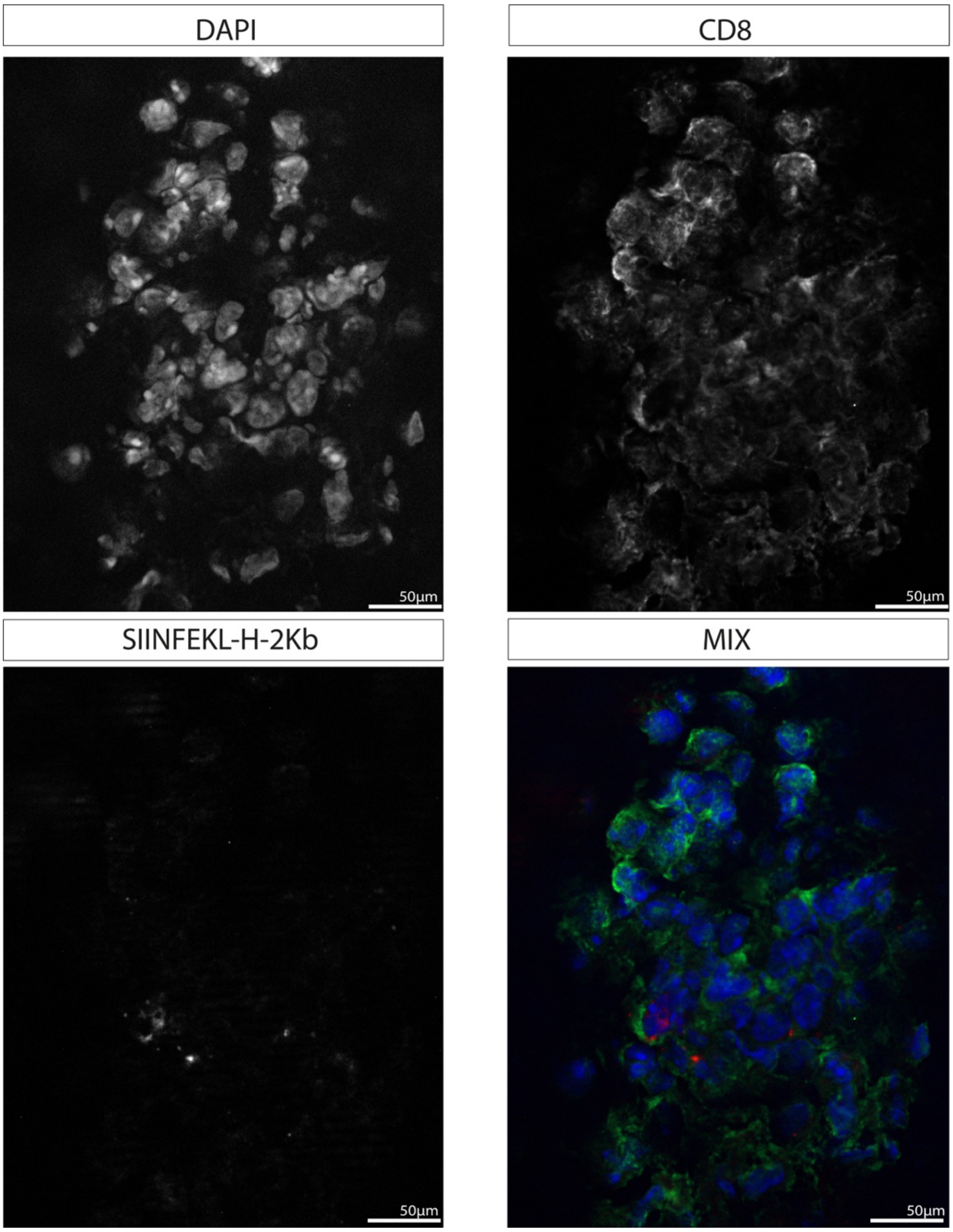

Supplement Figure 5: SIINFEKL-H-2Kb-CD8-DAPI Co-Staining. CD8 (green), DAPI (blue), SIINFEKL-H-2Kb (red). CD8 T cells were found to co-localize with SIINFEKL-H-2Kb in BL6-OVAV mice.

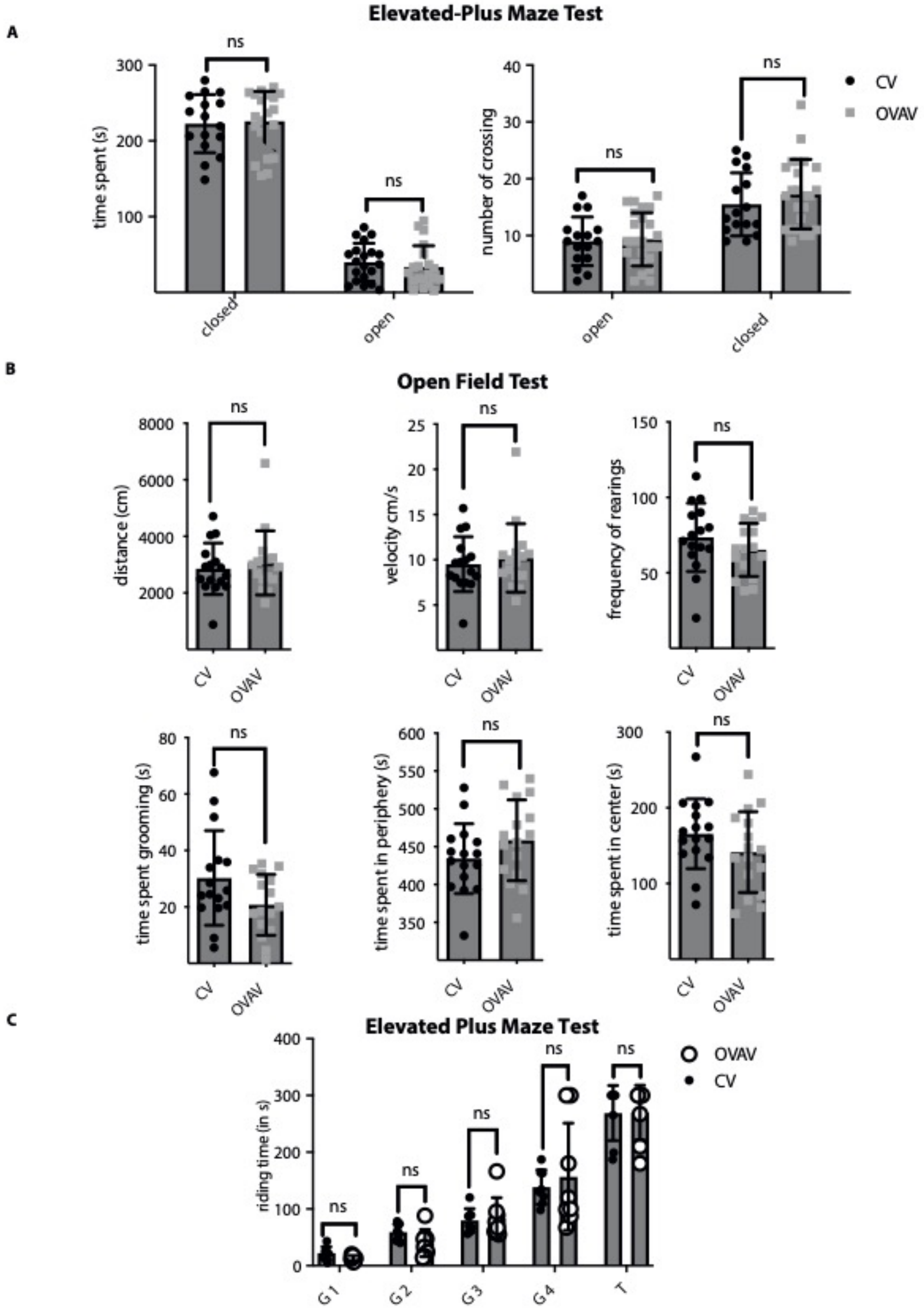
A
Elevated-Plus Maze Test
time spent (s)
300
200
100
closed
open
ns
number of crossing
40
30
20
10
CV
OVAV
B
Open Field Test
distance (cm)
8000
6000
4000
2000
velocity cm/s
25
20
15
10
5
frequency of rearings
150
100
50
time spent grooming (s)
80
60
40
20
time spent in periphery (s)
600
550
500
450
400
350
time spent in center (s)
300
200
100
C
Elevated Plus Maze Test
riding time (in s)
400
300
200
100
0
G1
G2
G3
G4
T

Supplement Figure 6: Behavior assessment in mice with cytotoxic T cell-mediated ALE. (A) Elevated Plus Maze one week after vector-based neuronal antigen transfer. The time the animals spent in the closed and open arms (N= 16 animals/group, $p = 0.84$) are not significantly different between SIINFEKL-CASAC immunized BL6-OVAV- (white bars) and BL6-CV- (black bars) injected mice. Mice of both groups spent significantly more time in the closed compared to the open arms ($p < 0.001$). **(B)** Open Field Test one week after vector-based neuronal antigen transfer. Behavioral parameters in the OF (distance, $p = 0.59$; velocity, $p = 0.58$; frequency of rearings $p = 0.25$; time spent grooming $p = 0.07$; time spent in periphery, $p = 0.18$; time spent in center, $p = 0.18$) are not significantly different between SIINFEKL-CASAC immunized BL6-OVAV- (white bars) and BL6-CV- (black bars) injected mice. **(C)** Rotarod one week after vector-based neuronal antigen transfer. Behavioral parameter in the rotarod test (drop-off time) is not significantly different between SIINFEKL-CASAC immunized BL6-OVAV- (white bars) and BL6-CV- (black bars) injected mice (N =16 animals/group, $p = 0.43$). Statistical significance was determined by Two-Way-ANOVA with Bonferroni post-hoc test in A and C and with Student's T test or Mann-Whitney-U test respectively in B.

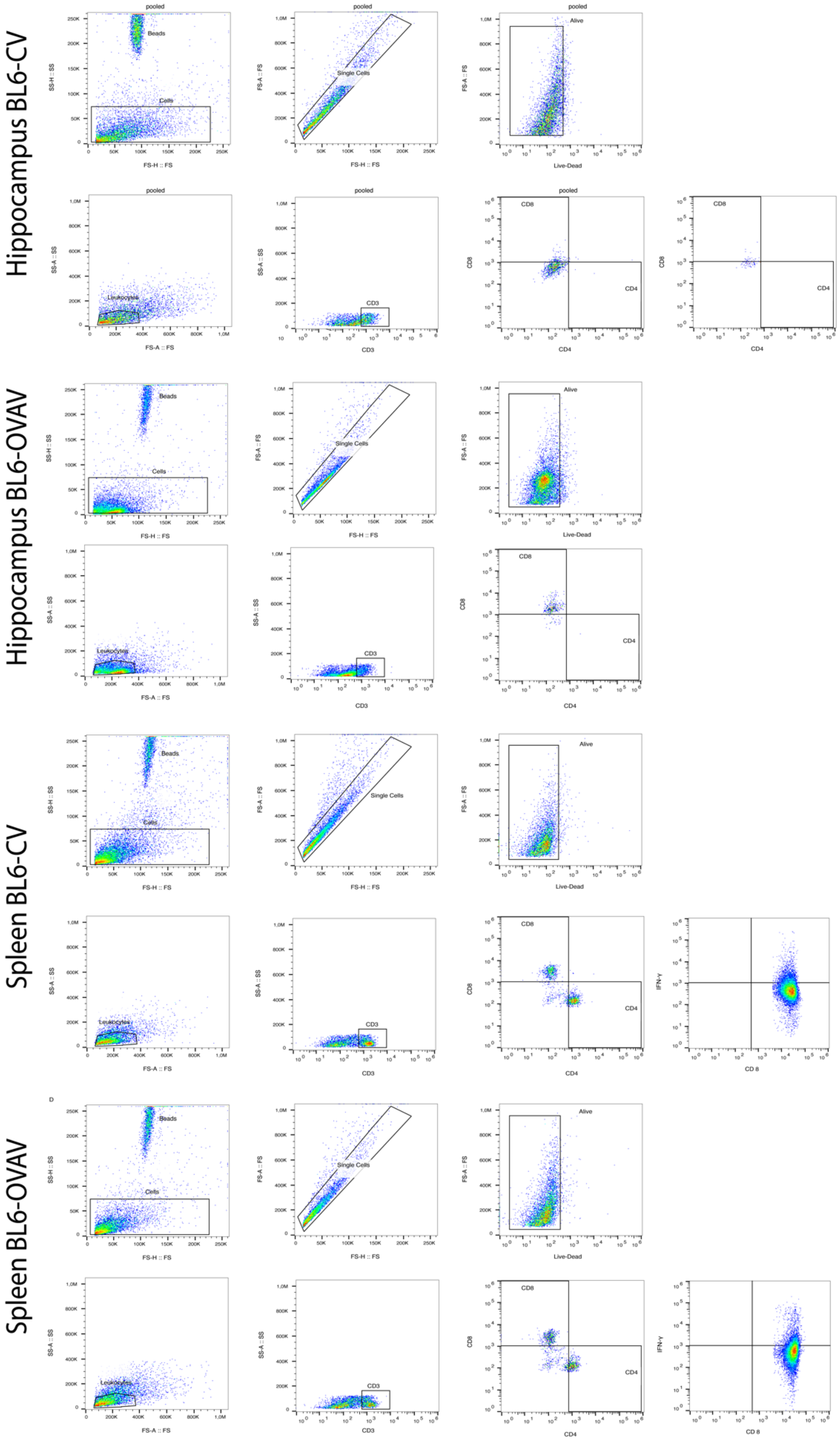

Hippocampus BL6-CV
Hippocampus BL6-OVAV
Spleen BL6-CV
Spleen BL6-OVAV
pooled
Beads
Cells
Single Cells
Alive
Leukocytes
CD3
CD8
CD4
SS-H :: SS
FS-H :: FS
FS-A :: FS
SS-A :: SS
Live-Dead
IFN-γ
CD 8

Supplement Figure 7: Flow cytometry gating strategy for brain and spleen CD8 T cells. Leukocytes were selected from a forward scatter area vs side scatter-area dot plot, and single cells were subsequently selected in a forward scatter-area vs forward scatter height dot plot. Then dead cells were excluded. Following that, T cells were selected by CD3+ expression and $CD8^{+}$and $CD4^{+}$ helper T cells were identified by a CD8 vs CD4 dot plot. To better visualize the gating strategy for CD8 T cells in the hippocampus of BL6-CV animals, 4 animals were pooled, T cells were rarely found in this group. Furthermore, exemplary dot plots for CD8-INF-γ+ spleenocytes following restimulation with SIINFEKL-peptide are provided.

ID	Age	Sex	Handed-ness	Disease duration at imaging	Clinical Symptoms	Compliance with diagnostic criteria	Mesial temporal TSPO-PET signal intensity (visual assessment)	Mesial temporal FLAIR-MRI abnormality (visual assessment)	Anterior temporal EEG abnormality (visual assessment)	Left mesial temporal cognitive dysfunction (z-score)	Right mesial temporal cognitive dysfunction (z-score)	Significant mesial temporal cognitive dissociation	Other autoimmune disorders, tumors	Pretreatment
1	63	m	right	0 month	generalized seizures, memory disturbance	possible ALE	left > right	left signal increase	none	yes (-1.08)	no (-0.04)	yes	MGUS	prednisolone (10 mg/day)
2	43	f	right	5 months	temporal lobe seizures, memory disturbance, affective disturbance	definite ALE	right > left	right signal increase	right slowing and sharp-slow-waves	no (1.73)	yes (-1.23)	yes	none	none
3	22	m	right	2 months	temporal lobe seizures, memory disturbance	possible ALE	left > right	left signal increase	left slowing	no (-0.25)	yes (-2.72)	yes	neurodermatitis	none
4	66	f	unknown	several months	temporal lobe seizures, polyneuropathy	definite ALE	left > right	left > right increase	left > right slowing, left sharp-slow-waves,	no (2.05)	no (1.89)	no	SCLC	paclitaxel, prednisolone (20 mg/day)
5	66	f	right	30 years	temporal lobe seizures	definite ALE	left = right	right > left signal increase	right slowing	no (-0.17)	no (-0.81)	no	Hashimoto thyroiditis, vitiligo	lamotrigine, brivaracetam, zonisamide, perampanel
6	63	m	right	several months	generalized seizures, memory disturbance	possible ALE	left > right	left > right signal increase	none	no (-0.57)	no (0.08)	no	none	none
7	50	f	right	4 years	temporal lobe seizures, affective disturbance	possible ALE	left ≥ right	left > right signal increase	left = right slowing	yes (-1.75)	yes (-2.33)	no	none	MTX, levetiracetam , oxcabazepine
8	51	f	right	24 years	temporal lobe seizures, memory disturbance, affective disturbance	definite ALE	left > right	left > right signal increase	left slowing, left > right sharp-waves	no (1.02)	not determined	not determined	Hashimoto thyroiditis	MMF, RTX, brivaracetam, lacosamide, clonazepam
9	29	m	right	3 years	temporal lobe seizures, generalized seizures	definite ALE	left > right	left > right signal increase	left slowing	no (1.02)	no (1.04)	no	vitiligo	none
10	59	f	right	4 years	temporal lobe seizures, generalized seizures memory disturbance, affective disturbance	definite ALE	left = right	left > right signal increase	Left = right slowing and sharp-slow-waves	no (-0.07)	yes (-1.41)	yes	thymoma, meningioma	lacosamide

Supplement Table 1: Demographics and clinical characteristics of patients

ID	Cell count (1/µl)	Total protein (mg/l)	Albumin ratio ($x10^{-3}$)	Blood-CSF-barrier dysfunction	Intrathecal IgG synthesis (%)	Intrathecal IgM synthesis (%)	Intrathecal IgA synthesis (%)	OCB type	Glucose ratio	Lactate (mmol/l)	Antibody-type	Specific IgG CSF	Specific IgG Serum	ASI
1	1	556	5.3	no	0	0	0	5	0.64	1.3	none	-	-	-
2	2	513	4.0	no	0	0	0	1	0.69	0.61	GAD65	1:10	1:100	37.3
3	1	431	2.8	no	0	0	0	1	0.73	1.57	none	-	-	-
4	18	675	9.3	yes	35	0	0	3	0.49	1.92	Hu<br>CV2<br>SOX1<br>ZIC4	64<br>580<br>416<br>336	357<br>4743<br>4488<br>3519	24.6<br>16.8<br>12.7<br>13.1
5	0	277	3.8	no	0	0	0	1	0.66	1.57	GAD65	1:1	1:1000	0.5
6	1	221	3.7	no	0	0	0	1	0.69	1.94	none	-	-	-
7	1	376	4.7	no	0	0	0	1	0.47	1.7	none	-	-	-
8	0	349	4.1	no	0	0	0	1	0.65	1.48	GAD65	1:100	1:3200	13.4
9	1	769	10.8	yes	0	0	0	1	0.67	1.28	GAD65	1:10	1:1000	1.9
10	1	411	3.7	no	0	0	0	1	0.65	1.65	GAD65	100	1:100	463.7

Supplement Table 2: Blood and CSF parameters of patients

Abbreviations: Albumin ratio= CSF/Plasma Albumin ratio, OCB = Oligoclonal band, Glucose ratio= CSF/plasma glucose, CV2= 66 kDa cytoplasmic protein (CRMP5) antibody, SOX1= antiglial nuclear antibody (AGNA), ZIC 4= Zinc Finger-Protein 4, ASI = antibody specificity index (ASI) = CSF/serum difference of antibody amounts per weight unit IgG.

Auxiliary Excel file with Raw Data
